# RBM15 drives bladder cancer progression through YTHDF2-dependent m6A-mediated regulation of ZO2

**DOI:** 10.1186/s13046-026-03684-9

**Published:** 2026-03-30

**Authors:** Yuhui He, Yanqing Gong, Yucai Wu, Shiming He, Yang Wang, Wenzhi Gao, Tai Tian, Xinyu Xu, Liqun Zhou, Zhenduo Shi, Conghui Han, Ninghan Feng, Jianfeng Wang, Xuesong Li

**Affiliations:** 1 Department of Urology, Peking University First Hospital, Beijing, 100034 China; 2 Institute of Urology, Peking University, Beijing, 100034 China; 3 Beijing Key Laboratory of Urogenital Diseases (Male) Molecular Diagnosis and Treatment Center, Beijing, 100034 China; 4 Department of Urology, China-Japan Friendship Hospital, Beijing, 100029 China; 5 Department of Urology, Jiangnan University Medical Center, Wuxi, 214122 China; 6 Department of Urology, Medical School of Nantong University, Wuxi No. 2 People’s Hospital, Wuxi, 214002 China; 7 Department of Urology, Xuzhou Central Hospital, Southeast University, Xuzhou, 221000 China; 8 Department of Urology, Xuzhou Clinical School of Xuzhou Medical University, Xuzhou, 221004 China

**Keywords:** Bladder cancer, RNA-binding motif protein 15, N6-methyladenosine, Zona Occludens 2, YTH N(6)-methyladenosine RNA binding protein 2, Malignant progression

## Abstract

**Background:**

Bladder cancer (BC) presents a major clinical challenge due to high recurrence and progression rates, highlighting the need for novel therapeutic targets. While the N6-methyladenosine (m6A) writer complex is broadly implicated, the specific function and regulatory mechanism of its adaptor protein RNA binding motif protein 15 (RBM15) remain poorly defined in BC. This study reveals the oncogenic role of RBM15 in BC and its m6A-dependent regulatory axis, providing a new rationale for targeted intervention.

**Methods:**

The clinical relevance of RBM15 was established by assessing its expression and prognostic significance in public datasets and a large clinical cohort. The biological function of RBM15 and its effect on global m6A methylation were subsequently investigated using a comprehensive suite of in vitro assays and in vivo models. We performed integrated multiomics analyses (RNA-seq, m6A-seq, and RIP-seq) and validated the underlying molecular mechanisms by performing additional targeted assays to elucidate the downstream regulatory network. Finally, the therapeutic potential of targeting this axis was validated in preclinical models using the METTL3 catalytic inhibitor STM2457.

**Results:**

RBM15 was significantly upregulated in BC, and its elevated expression served as an independent predictor of a poor prognosis. Functionally, RBM15 increased global m6A levels and promoted the malignant progression of BC cells both in vitro and in vivo; these oncogenic effects were reversed upon RBM15 knockdown. Mechanistically, RBM15 bound Zona Occludens 2 (ZO2) mRNA and recruited the methyltransferase 3–methyltransferase 14–Wilms’ tumor 1-associating protein (METTL3-METTL14-WTAP) methyltransferase complex to increase the level of the m6A modification on the ZO2 mRNA. This modification was recognized by YTH N(6)-methyladenosine RNA binding protein 2 (YTHDF2) to accelerate ZO2 mRNA decay. Although ZO2 expression was globally reduced, its preferential nuclear accumulation was increased, which promoted Snail expression and accelerated malignant progression. The METTL3 catalytic inhibitor STM2457 suppressed BC growth and lung metastasis by targeting the METTL3/RBM15/ZO2 axis, with no observable toxicity.

**Conclusions:**

RBM15 acts as an oncogenic driver in BC by facilitating the m6A-dependent degradation of ZO2 mRNA via the recruitment of the METTL3 complex and recognition by YTHDF2. Targeting this METTL3/RBM15/ZO2 axis with STM2457 represents a promising therapeutic strategy for BC.

**Supplementary Information:**

The online version contains supplementary material available at 10.1186/s13046-026-03684-9.

## Background

Bladder cancer (BC), the ninth most common malignancy globally, caused ~ 614,000 new cases and 220,000 deaths in 2022 [1]. BC is classified as non-muscle-invasive BC (NMIBC, 70% of cases) or muscle-invasive BC (MIBC), and NMIBC recurs in 30–80% of patients within 5 years, with 45% of patients progressing to MIBC [2–4]. The prognosis of patients with MIBC remains poor (5-year overall survival: untreated 5% vs. treated 48%) [5], underscoring the urgent need for mechanistic insights and biomarkers.

In addition to genetic drivers [6, 7], epitranscriptomic regulation via the N6-methyladenosine (m6A) modification critically influences BC pathogenesis [8]. This prevalent messenger ribonucleic acid (mRNA) modification, governed by writers/erasers/readers, regulates RNA processing [9]. While methyltransferase 3 (METTL3) is an established oncogenic writer in BC [10–12], the role of its crucial adapter, RNA-binding motif protein 15 (RBM15), remains largely uncharacterized in this context. Although RBM15 has been implicated as an m6A-driven oncogene in several solid tumors, its mechanism of action appears to be highly context specific (Table S1). For instance, in laryngeal squamous cell carcinoma (LSCC), RBM15 promotes tumorigenesis by stabilizing the TMBIM6 mRNA, whereas in hepatocellular carcinoma (HCC), it increases YES1 transcript stability [13, 14]. A consistent pattern emerging from these studies is that the RBM15-mediated m6A modification leads to the stabilization of its target transcripts. However, whether this prosurvival paradigm applies to BC remains unknown and represents a critical gap in our understanding.

In this study, we elucidated a novel and paradoxical mechanism. We show that RBM15 acts as a pivotal oncogenic driver in BC by recruiting the m6A writer complex to the Zona Occludens 2 (ZO2) mRNA, a previously unidentified target. Strikingly, in direct contrast to prior studies, this activity orchestrates the m6A hypermethylation and subsequent YTH N(6)-methyladenosine RNA binding protein 2 (YTHDF2)-dependent degradation of the ZO2 mRNA, thereby promoting malignant progression. Our findings not only establish the METTL3/RBM15/ZO2 axis as a novel vulnerability in BC but also underscore the potential for precision therapy through context-specific m6A modulation.

## Methods

### Bioinformatics analysis

Public RNA-sequencing data were sourced from The Cancer Genome Atlas Bladder Urothelial Carcinoma dataset (TCGA-BLCA) (*n* = 406 tumor samples) and the Genotype–Tissue Expression (GTEx) project (*n* = 21 normal bladder samples) [15, 16]. Additionally, microarray data comprising 165 primary BC samples, 23 recurrent BC samples, and 10 normal bladder tissue samples (Batch: GPL6480) were obtained from the Gene Expression Omnibus (GEO) under accession ID GSE13507. For inclusion, samples were required to be derived from primary tumors and have available survival and clinical staging information. The expression data were normalized using the ComBat algorithm from the sva R package to mitigate batch effects between the TCGA and GTEx cohorts. A differential expression analysis was performed using the DESeq2 R package, with thresholds set at an absolute log2 fold change (|log2FC|) > 1 and an adjusted *p* value (corrected with the false discovery rate, FDR) < 0.05. Survival analyses were conducted using the Kaplan‒Meier method with log-rank tests. The prognostic significance of RBM15 was further validated using Gene Expression Profiling Interactive Analysis 2 (GEPIA2) [17]. The m6A2Target database was used to predict potential m6A-modified targets [18].

### Patients and clinical specimens

A BC tissue microarray comprising 137 specimens with paired normal tissues (Peking University First Hospital, 2002–2012) was analyzed using clinicopathological data from medical records, including 114 males and 23 females (mean age 64.6 years) stratified by T stage: T1 (*n* = 39), T2 (*n* = 40), T3 (*n* = 26), and T4 (*n* = 32). Follow-up at 6 years (2014) revealed 129/137 patients (94% retention) and 77 survivors (59% survival), decreasing to 94/137 patients (68% retention) and 32 survivors (34% survival) by 13 years (2021). The demographic characteristics are shown in Table S2. This study was approved by the Ethics Committee of Peking University First Hospital (Approval No. 2025R0010-0002), with written informed consent obtained from all participants.

### Cell culture and conditions

Human 293T, T24, and 5637 cell lines were obtained from the American Type Culture Collection (ATCC, Manassas, VA, USA). The immortalized human urothelial cell line SV-HUC-1 was obtained from Procell Life Science (Wuhan, China), and the murine BC cell line MB49 was obtained from Patone Biotech (Shanghai, China). All the cell lines underwent short tandem repeat (STR) authentication and were maintained as specified in Table S3.

### Patient-Derived Organoids (PDOs)

Freshly resected BC tissues were obtained from three patients with newly diagnosed BC at Peking University First Hospital after obtaining written informed consent and ethical approval (Approval No. 2022-279-002). The tissues were minced and digested in a solution containing collagenase II (1 mg/mL; Invitrogen, Carlsbad, CA, USA, 17101015) and dispase II (1 mg/mL; Invitrogen, 17105041) for 1 h at 37 °C. The resulting cell suspension was filtered through a 70-µm cell strainer, and the pellet was resuspended in Matrigel (Corning, Corning, NY, USA, 354234) and seeded in 24-well plates. Organoids were cultured in advanced DMEM/F12 (Invitrogen, 12634010) supplemented with B27 (Invitrogen, 17504044), N2 (Invitrogen, 17502048), 100 ng/mL Noggin (PeproTech, Cranbury, NJ, USA, 120–10 C), 50 ng/mL EGF (PeproTech, AF-100-15), and 10 µM Y-27,632 (Selleck, Houston, TX, USA, S1049). For drug treatment, established organoids were treated with STM2457 (Selleck, S8809) for 72 h before analysis.

### Quantitative Real-time Polymerase Chain Reaction (qPCR)

Total RNA was extracted from tissues or cell lines using TRIzol™ reagent (Invitrogen, 15596026). Complementary deoxyribonucleic acid (cDNA) was synthesized using FastKing RT SuperMix (TIANGEN, Beijing, China, KR118) according to the manufacturer’s protocol. qPCR was performed in triplicate using TransStart Green qPCR SuperMix (TIANGEN, AQ142) on a QuantStudio 5 Real-Time PCR System (Applied Biosystems, Foster City, CA, USA). The expression levels were normalized to those of GAPDH and calculated using the 2^−ΔΔCt^ method. All primer sequences are listed in Table S4.

### Western Blot (WB)

Proteins were extracted using NP-40 lysis buffer (Beyotime, Shanghai, China, P0013F) supplemented with a protease and phosphatase inhibitor cocktail (BIMAKE, Houston, TX, USA, B14001). Protein concentrations were quantified using an Enhanced BCA Protein Assay Kit (Beyotime, P0009). WB was performed using established protocols [19]. Details and dilutions of the primary and secondary antibodies are specified in Table S5.

### Nuclear and cytoplasmic fractionation

Nuclear and cytoplasmic proteins were separated using a Nuclear and Cytoplasmic Protein Extraction Kit (Beyotime, P0027) according to the manufacturer’s instructions. Briefly, cells were lysed in cytoplasmic extraction buffer, and the supernatant containing the cytoplasmic fraction was collected after centrifugation. The remaining nuclear pellet was then lysed in nuclear extraction buffer. The protein fractions were subsequently analyzed by WB.

### Immunohistochemistry (IHC)

For IHC, deparaffinized sections were subjected to antigen retrieval in citrate buffer (pH 6.0) at 95 °C for 20 min. Endogenous peroxidase activity was blocked with 3% H₂O₂, and nonspecific binding was blocked with 5% goat serum. The sections were incubated overnight at 4 °C with primary antibodies (Table S5), followed by development using the Polink-2 Plus HRP Detection System (ZSGB-Bio, PV-9001). Scoring was performed using an immunoreactivity scoring (IRS) system [20], which multiplied the staining intensity (0–3) by the percentage of positive cells (0–4). Two board-certified uropathologists, who were blinded to the clinical data, independently assessed all the sections.

### Immunofluorescence (IF) staining

For IF, cells grown on coverslips were fixed with 4% paraformaldehyde (PFA) for 15 min, permeabilized with 0.1% Triton X-100 (Beyotime, ST797) for 10 min, and blocked with 3% bovine serum albumin (BSA; Beyotime, ST2249) for 1 h. The cells were incubated with primary antibodies (Table S5) overnight at 4 °C, followed by an incubation with Alexa Fluor-conjugated secondary antibodies for 1 h at room temperature (RT). The nuclei were counterstained with DAPI (Beyotime, C1005, 1 µg/mL) for 10 min before the cells on the coverslips were mounted. Images were acquired using a Leica TCS SP8 confocal microscope.

### Construction of gene knockdown and overexpression cell lines

Lentivirus-based short hairpin RNA (shRNA)-mediated knockdown or pLVX-IRES-overexpressing cell lines were generated. Target-specific shRNAs were designed using the GPP Web Portal and validated by BLAST (Table S6). For overexpression, coding DNA sequence (CDS) or 3’ untranslated region (3’UTR) fragments were amplified using primers designed with Primer Premier 5.0 and cloned and inserted into the respective vectors (Table S6). All the constructs were verified by restriction enzyme digestion, gel purification (QIAGEN, Hilden, Germany, 28704), and Sanger sequencing. Lentiviruses were packaged in 293T cells using psPAX2 and pMD2.G plasmids. Target cells were infected in the presence of polybrene, followed by selection with puromycin or blasticidin. The knockdown or overexpression efficiency was confirmed by qPCR and WB and compared with cells infected with lentiviruses carrying a nontargeting shRNA or empty vector as controls. Validated cell lines were cryopreserved for future use.

### Generation of gene knockout cell lines

CRISPR/Cas9-mediated knockout was performed using the lentiCRISPRv2 plasmid (Addgene, Watertown, MA, USA, 52961). Single guide RNAs (sgRNAs), designed using CRISPOR, were cloned and inserted into the BsmBI sites of the vector (Table S7). Lentiviruses were produced in HEK293T cells by cotransfecting the sgRNA plasmid with psPAX2 and pMD2.G using Lipofectamine 3000 (Invitrogen). Target cells were transduced with the lentiviruses at a multiplicity of infection (MOI) of 5 with 8 µg/ml polybrene for 24 h, followed by selection with 2 µg/ml puromycin for 7 days. Single-cell clones were isolated, and the editing efficiency was assessed by performing a T7 endonuclease I (T7E1) assay on PCR-amplified target regions. Knockout was confirmed by WB. Validated clones were cryopreserved.

### Cell proliferation, migration, and invasion assays

Cell proliferation was assessed using a Cell Counting Kit-8 (CCK-8; KeyGEN, Nanjing, China, KGA317). Cells were seeded at a density of 1,000 cells/well in 96-well plates. At specified time points, 110 µL of CCK-8 solution (1:10 in serum-free medium) was added, and the absorbance at 450 nm was measured after a 2-hour incubation using a microplate reader (Beckman Coulter, Brea, CA, USA).

Migration and invasion assays were performed using Transwell chambers (Corning, Corning, 3422). For invasion assays, the chambers were precoated with Matrigel (Corning, 354262). Cells (1–4 × 10⁴/well) were seeded in the upper chamber in serum-free medium, with the lower chamber containing medium supplemented with 10% FBS. After 24 h (migration) or 48 h (invasion), nonmigrated cells were removed, and migrated cells were fixed, stained with 0.1% crystal violet, and quantified in three random 100× fields.

### Drug sensitivity assay

Cells were seeded in 96-well plates at a density of 5,000 cells/well and allowed to adhere overnight. The following day, the cells were treated with increasing concentrations of the METTL3 inhibitor STM2457 (Selleck, S8809). After 72 h of incubation, cell viability was measured using the CCK-8 assay as described above. The half-maximal inhibitory concentration (IC50) was calculated by fitting the dose‒response data to a four-parameter logistic curve using GraphPad Prism 9 (GraphPad Software, San Diego, CA, USA).

### Animal experiments

All animal procedures were approved by the Peking University First Hospital Animal Ethics Committee (J2021115) and conducted in strict accordance with the 3R principles (replacement, reduction, and refinement). Sample sizes were determined based on a priori power analysis using data from preliminary experiments. Assuming a large effect size (d ≥ 1.2) with a standard deviation of 30% of the mean, we calculated that a minimum of 4–6 mice per group would provide > 80% power at an alpha level of 0.05. Accordingly, 4 mice per group were used for the low-variability subcutaneous models, while 4 or 6 mice per group were allocated to the metastasis and orthotopic models to account for potentially higher biological variability. Animals were randomly assigned to treatment or control groups using a random number generator. All tumor measurements and bioluminescence imaging were performed by investigators who were blinded to the treatment allocation.

For subcutaneous xenografts, 5 × 10⁶ cells in Matrigel were injected into 3-week-old male BALB/c nude mice. The tumor volume [(length×width²)×π/6] was measured every 3 days. For tail vein metastasis models, 1 × 10⁵ luciferase-expressing cells were injected, and the metastatic burden was monitored weekly via bioluminescence imaging (IVIS Spectrum, PerkinElmer). For the orthotopic models, 5 × 10⁴ luciferase-tagged cells were injected into the bladder wall of 4-week-old male nude mice after laparotomy and bladder scraping.

### RNA sequencing (RNA-seq) and functional enrichment analysis

Total RNA was extracted from three biological replicates of RBM15-silenced and control T24 cells. Libraries were prepared using the TruSeq Stranded mRNA Kit (Illumina, San Diego, CA, USA, 20020595) and sequenced (PE150) on the NovaSeq 6000 platform. Raw reads were trimmed (Trimmomatic), aligned to the GRCh38 human genome (STAR v2.7), and quantified (featureCounts v2.0). Differentially expressed genes (DEGs) were identified using DESeq2 (|log2FC| > 1, padj. < 0.05). Functional annotation was performed using clusterProfiler with KEGG (2023Q1), and gene set enrichment analysis (GSEA) was conducted using the MSigDB Hallmark v7.5.1 gene sets.

### M6A RNA immunoprecipitation sequencing (MeRIP-seq)

Total RNA was fragmented to ~ 100 nt and immunoprecipitated with an anti-m6A antibody (Synaptic Systems, Goettingen, Germany, 202003) using a Magna MeRIP Kit (Millipore, Burlington, MA, USA, 17-10499). Enriched RNA was captured on Protein A/G magnetic beads (Thermo Fisher Scientific, Waltham, MA, USA; 88845). Libraries were prepared using the NEBNext Ultra II RNA Library Prep Kit (NEB, Ipswich, MA, USA, E7770) and sequenced using the Illumina NovaSeq 6000 platform. Differentially abundant m6A peaks were identified using the exomePeak2 R package with standard parameters (*p* < 1e-05) and defined as having a |log2FC| > 1 and an FDR < 0.05.

### RNA immunoprecipitation sequencing (RIP-seq)

RIP-seq was performed using an anti-RBM15 antibody (Abcam, Cambridge, UK, ab244250) or IgG as a control (Cell Signaling Technology, Danvers, MA, USA, 2729 S). The fragmented RNA was immunoprecipitated as described for MeRIP-seq. RBM15 binding sites (peaks) were identified using Piranha (v1.2.1) with a zero-truncated negative binomial distribution model, a bin size of 50 nt, and a *p* value threshold of 0.05, with the IgG control used as the background.

### RNA dot blot

Global m6A levels were assessed via dot blotting using an anti-m6A antibody (Abcam, ab284130). Total RNA from cells/tissues was serially diluted (2–0.25 µg/dot), denatured (95 °C, 3 min), snap-frozen, and spotted onto nylon membranes (Beyotime, China, FFN10; 2 µL/dot). After UV crosslinking (1 h), the membranes were blocked (3% BSA), incubated with the primary antibody (4 °C/overnight) followed by the HRP-conjugated secondary antibody (RT, 1 h), developed with ECL substrate, and counterstained with 1% methylene blue for normalization. Total RNA was serially diluted and spotted onto the membrane to ensure equal loading. After immunodetection with an anti-m6A antibody, the membrane was stained with 1% methylene blue to visualize total RNA, which served as a loading control.

### M6A RNA immunoprecipitation–qPCR (m6A-IP–qPCR)

m6A-modified RNA was enriched using the m6A RNA Methylation Enrichment Kit (Epigentek, China, A-P-9018). Total RNA (2 µg) was fragmented, immunoprecipitated with m6A antibody-conjugated beads in capture buffer (RT, 90 min), treated with nucleases and then washed. Bound RNA was eluted with Proteinase K (55 °C, 15 min), precipitated with ethanol, and analyzed using qPCR. Input controls were processed without IP. The relative enrichment of m6A-modified RNA for specific targets was calculated using the percent input method, where the amount of immunoprecipitated RNA was normalized to the amount of input RNA for each primer set.

### RNA stability assay

Cells were treated with 5 µg/ml actinomycin D (Aladdin, Shanghai, China, A114787). RNA was isolated at 0, 3, 6, and 9 h, and target transcript levels were quantified by qPCR to determine the mRNA half-life.

### RNA immunoprecipitation–qPCR (RIP–qPCR)

RNA‒protein interactions were analyzed using a Magna RIP Kit (Millipore, USA; 17–700). Lysates from 2 × 10⁷ cells were incubated with antibody-conjugated beads (5 µg antibody/100 µL beads, 30 min RT, overnight at 4 °C). After being washed, the bound RNA was extracted with phenol‒chloroform–isoamyl alcohol (125:24:1) and precipitated with ethanol. The input lysate (10%) served as a control. RBM15 binding sites were identified using Piranha, a tool specifically designed for RNA‒protein interaction data, with a zero-truncated negative binomial model (*p* < 0.05).

### Chromatin immunoprecipitation (ChIP)-seq and ChIP‒qPCR

For ChIP-seq, cells were cross-linked with 1% formaldehyde, and the chromatin was sonicated to produce fragments with an average size of 200–500 bp. Immunoprecipitation was performed overnight at 4 °C using an anti-ZO2 antibody (Proteintech, Rosemont, IL, USA, 21883-1-AP) or an IgG control (Cell Signaling Technology, 2729 S). After the reversal of cross-links and DNA purification, the libraries were prepared and sequenced. Peak calling was performed using MACS2 (v2.2.7) with a *q* value cutoff of 0.05.

For ChIP‒qPCR, immunoprecipitated DNA was analyzed by qPCR using primers specific to the SNAI1 promoter and a negative control intergenic region. The primer sequences are listed in Table S8. Enrichment was calculated relative to the input control.

### Chromatin isolation by RNA purification (ChIRP)

Chromatin-associated RNA was isolated using a ChIRP Kit (BersinBio, Guangzhou, China, bes5104-2) with nine ZO2-targeting biotinylated probes (Table S9). Formaldehyde-crosslinked chromatin from 6 × 10⁷ cells was sonicated (30% amplitude, 5 s on/3 s off, 40 min), hybridized with probes (37 °C), and captured by streptavidin beads. After being washed, the RNA‒protein complexes were eluted separately for qPCR/WB analyses.

### Coimmunoprecipitation (Co-IP)

Co-IP was performed using a Pierce Magnetic IP/Co-IP Kit (Thermo Fisher Scientific, 88804). The cell lysates (1 mg protein) were incubated with 4 µg of the indicated antibody overnight at 4 °C. Immune complexes were captured, washed, and eluted according to the manufacturer’s protocol, followed by WB analysis. The details on the antibodies are provided in Table S5.

### Statistical analysis

All quantitative data are presented as the means ± standard deviations (SDs) from at least three independent experiments. Statistical analyses were performed using SPSS 23.0 (IBM, Armonk, NY, USA) and R 3.4.1. For two-group comparisons, a two-tailed Student’s t test was used. For comparisons involving more than two groups, one-way analysis of variance (ANOVA) followed by Tukey’s post hoc test were employed. For cell proliferation assays measured over time, two-way ANOVA was used. Pearson’s correlation coefficients were calculated to assess linear relationships. The survival analysis was conducted using the Kaplan‒Meier method with the log-rank test. Significance levels are denoted as *p < 0.05, **p < 0.01, and ***p < 0.001; ns, not significant.

## Results

### RBM15 is upregulated in BC and correlates with a poor prognosis

**Fig. 1:**
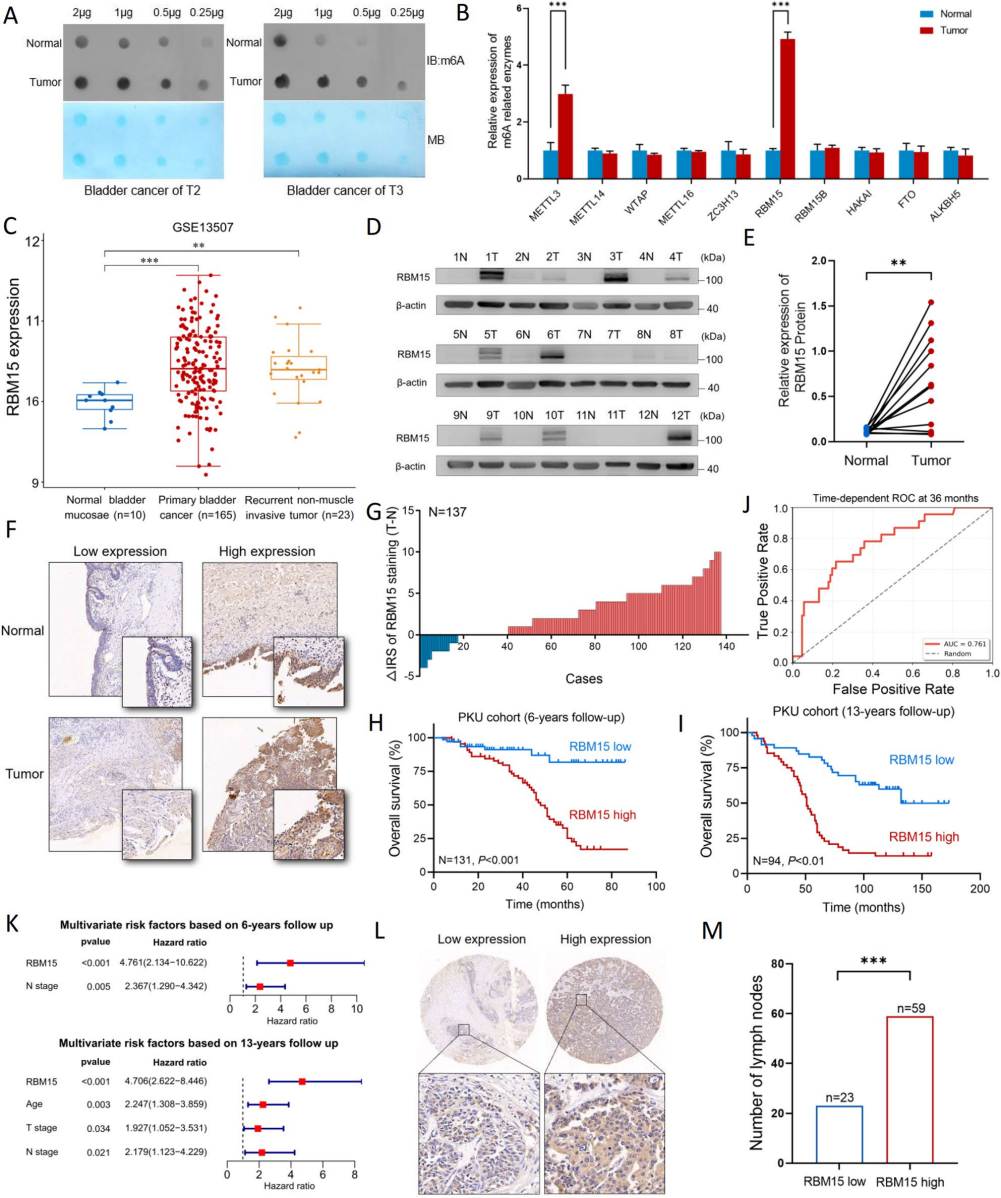
RBM15 is upregulated in BC and correlates with a poor prognosis.** A** RNA dot blot analysis showing elevated global m6A levels in tumor tissues compared with paired adjacent normal tissues from patients with T2 and T3 stage BC. Methylene blue (MB) staining served as a loading control. **B** qPCR analysis of the mRNA expression of key m6A methyltransferases and demethylases in BC and normal tissues. **C** RBM15 mRNA expression in the GSE13507 cohort, comparing normal bladder mucosae (*n* = 10), primary BC (*n* = 165), and recurrent non-muscle-invasive tumors (*n* = 23). **D** WB analysis of RBM15 protein levels in 12 paired BC tumor (**T**) and adjacent normal (**N**) tissues. **E** Quantification of RBM15 protein expression from the WBs in (**D**). **F** Representative images of IHC staining showing low and high expression of RBM15 in paired normal and tumor tissues. Scale bars are indicated. Left panel: 100 × (10× objective × 10× ocular), right panel: 400 × (10× objective × 40× ocular). **G** Distribution of RBM15 IHC staining scores in the 137-patient cohort. **H**,** I** Kaplan-Meier curves for overall survival based on RBM15 expression levels in the PKU cohort with (**H**) 6-year and (**I**) 13-year follow-up. **J** Time-dependent ROC curve analysis of the RBM15-based prognostic model. **K** Forest plots of the multivariate Cox regression analysis of 6-year and 13-year overall survival, identifying RBM15 as an independent prognostic factor. **L** Representative images of IHC staining showing RBM15 expression in metastatic lymph nodes. Upper panel, 40 × (10× objective × 4× ocular); lower panel, 400 × (10× objective × 40× ocular). **M** Semiquantitative IRS of RBM15 in metastatic lymph nodes (*n* = 82) based on five randomly selected metastatic tumor regions. Upper panel: 100 × (10× objective × 10× ocular), lower panel: 400 × (10× objective × 40× ocular). **p < 0.01 and ***p < 0.001

We investigated the role of the m6A modification in BC by first assessing global m6A levels in paired tumor and adjacent normal tissues. An RNA dot blot analysis revealed a significant increase in global m6A methylation in tumor tissues from patients with both T2 and T3 stage BC (Fig. 1A). We profiled the mRNA expression of key m6A methyltransferases and demethylases to identify the potential drivers of this hypermethylation. While the role of METTL3 in BC has been partially established [11, 12], our qPCR results showed that RBM15 was the most significantly upregulated methyltransferase in BC tissues compared to normal urothelium, suggesting that it may be a novel and critical driver of m6A dysregulation in this context (Fig. 1B).

These findings were further validated in large-scale public cohorts. An analysis of The Cancer Genome Atlas (TCGA) and Genotype-Tissue Expression (GTEx) datasets confirmed the marked overexpression of RBM15 in BC tissues compared with normal bladder mucosae (Fig. S1A). Moreover, data from the GSE13507 cohort demonstrated that RBM15 expression was significantly increased in both primary and recurrent nonmuscle-invasive tumors compared with normal bladder tissue (Fig. 1C). At the protein level, WB analysis of 12 paired BC and normal tissues showed that RBM15 expression was upregulated in 8 of the 12 tumor samples, with quantification confirming a significant overall increase (Fig. 1D and E). Consistent with these findings, IHC performed on a cohort of 137 paired tumor and normal tissues revealed high RBM15 expression in 71% of the BC cases (Fig. 1F and G).

We next evaluated the prognostic significance of RBM15 expression. In TCGA cohort, high RBM15 expression was associated with significantly shorter disease-free survival (DFS) (Fig. S1B). In our own PKU cohort, the Kaplan‒Meier analysis indicated that patients with high RBM15 expression experienced markedly shorter overall survival (OS) at both the 6-year and 13-year follow-ups (Fig. 1H and I). The predictive accuracy of the RBM15-based model was assessed by performing a time-dependent ROC curve analysis, which showed that the AUC increased from 0.616 at 12 months to 0.707 at 24 months and 0.761 at 36 months, indicating stable and improved predictive power over time (Fig. 1J; Fig. S1C). We performed a multivariate Cox regression analysis to determine the independent prognostic value of this model. After adjusting for confounding clinical variables, RBM15 expression emerged as a robust and independent predictor of a poor prognosis for both 6-year and 13-year OS (Fig. 1K, Fig. S1D, S1E). Analyses of patients stratified by TNM stage and tumor grade revealed that the prognostic value of RBM15 was most pronounced in advanced and high-grade BC subgroups (Tables S10 and S11). Furthermore, decision curve analysis (DCA) demonstrated that the RBM15-based model provided substantial net clinical benefit across a wide range of probability thresholds (30–50%) (Fig. S1F). Notably, at the 50% threshold, the model offered a net benefit of + 0.3953 compared with the “treat all” strategy, suggesting that its use could prevent unnecessary treatment in approximately 40% of patients while ensuring that high-risk individuals are appropriately managed (Table S12). Additional validation in the GSE31684 cohort (*n* = 88) confirmed that high RBM15 expression significantly correlated with shorter median survival (Fig. S1G). The multivariate Cox regression analysis indicated that high RBM15 expression was an independent predictor of a 4.6-fold increased risk of death, highlighting its critical role in clinical risk stratification (Table S13). Finally, IHC analysis of metastatic lymph nodes revealed that RBM15 was overexpressed in 72% of samples, strongly linking its expression to disease dissemination (Fig. 1L and M).

Collectively, these data establish RBM15 as an independent prognostic biomarker in BC, where its upregulation is strongly associated with aggressive disease, metastatic dissemination, and poor clinical outcomes.

### RBM15 drives malignant progression and m6A hypermethylation in BC in vitro

**Fig. 2:**
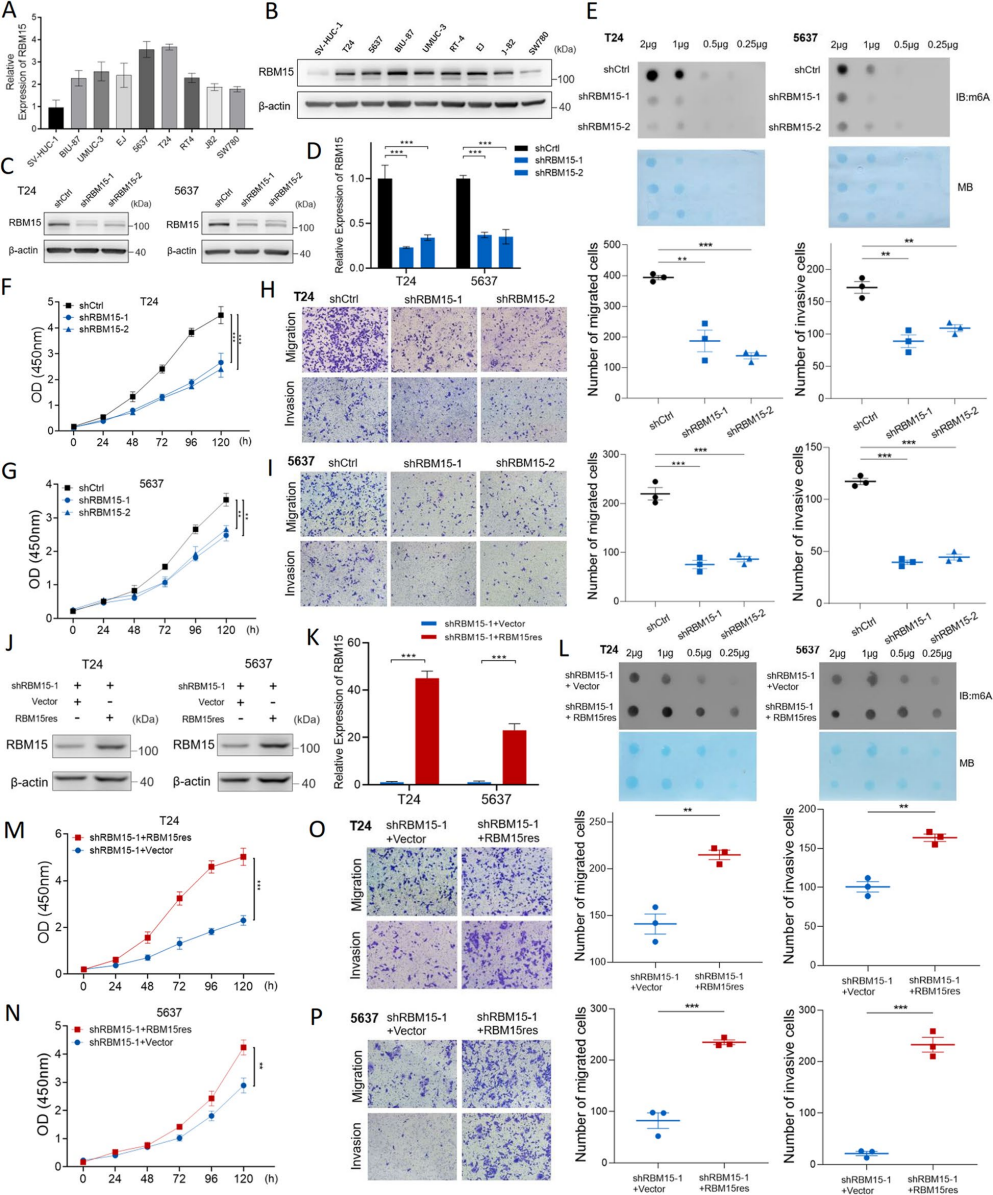
RBM15 drives m6A hypermethylation and the malignant progression of BC in vitro. **A**,** B** qPCR (**A**) and WB (**B**) analyses showing upregulated RBM15 expression in a panel of BC cell lines compared with the immortalized urothelial cell line SV-HUC-1. **C**,** D** Efficient knockdown of RBM15 in T24 and 5637 cells using two independent shRNAs (shRBM15-1 and shRBM15-2), as confirmed by WB (**C**) and qPCR (**D**). **E** RNA dot blot analysis showing a reduction in global m6A methylation levels upon RBM15 knockdown. Methylene blue (MB) staining served as a loading control. **F**,** G** CCK-8 assays showing that RBM15 knockdown significantly inhibited the proliferation of T24 (**F**) and 5637 (**G**) cells. **H**,** I** Transwell assays demonstrating that RBM15 knockdown suppressed the migration and invasion of T24 (**H**) and 5637 (**I**) cells. Representative images and the results of the quantitative analysis are shown. Scale bar, 100 × (10× objective × 10× ocular). Three randomly selected fields per sample (100× magnification) were quantified. **J**,** K** WB (**J**) and qPCR (**K**) confirmation of successful RBM15 re-expression (RBM15res) in RBM15-knockdown cells. **L** RNA dot blot analysis showing that the re-expression of RBM15 rescued global m6A methylation levels. **M**,** N** CCK-8 assays showing that RBM15 re-expression reversed the inhibition of the proliferation of T24 (**M**) and 5637 (**N**) cells. **O**,** P** Transwell assays showing that RBM15 re-expression restored the migratory and invasive capacities of T24 (**O**) and 5637 (**P**) cells. Scale bars are the same as in H–I. **p < 0.01 and ***p < 0.001

We investigated the functional role of RBM15 in vitro by first profiling its expression across a panel of human BC cell lines. Consistent with our findings from patient tissues, both qPCR and WB analyses revealed that RBM15 expression was markedly upregulated at both the mRNA and protein levels in multiple BC cell lines compared with that in the immortalized normal urothelial cell line SV-HUC-1. The T24 and 5637 cell lines, which exhibited the highest endogenous RBM15 expression, were selected for subsequent loss-of-function studies (Fig. 2A, B).

We established stable RBM15-knockdown T24 and 5637 cells using two independent shRNAs (shRBM15-1 and shRBM15-2) to determine its function. The knockdown efficiency was confirmed at both the transcript and protein levels (Fig. 2C, D). We then assessed the effect of RBM15 depletion on global m6A methylation. As hypothesized, the RNA dot blot analysis revealed a significant reduction in total m6A levels in RBM15-silenced cells, directly indicating that RBM15 is a key contributor to m6A installation in BC (Fig. 2E).

Functionally, the depletion of RBM15 markedly suppressed the malignant phenotypes of BC cells. CCK-8 assays demonstrated that RBM15 knockdown significantly inhibited the proliferation of both T24 and 5637 cells (Fig. 2F, G). Furthermore, Transwell assays revealed that the migratory and invasive capabilities of these cells were substantially impaired upon RBM15 silencing (Fig. 2H, I).

We performed rescue experiments by re-expressing an shRNA-resistant form of RBM15 (RBM15res) in the knockdown cells to confirm that the observed phenotypes were specifically due to RBM15 depletion (Fig. 2J, K). The restoration of RBM15 expression not only rescued the global m6A methylation levels (Fig. 2L) but also fully reversed the inhibitory effects on cell proliferation (Fig. 2M, N), migration, and invasion (Fig. 2O, P).

Taken together, these results unequivocally show that RBM15 functions as a critical oncogenic driver in BC by promoting m6A hypermethylation and sustaining malignant cell behaviors.

### RBM15 drives BC growth and metastasis and promotes m6A hypermethylation in vivo

**Fig. 3:**
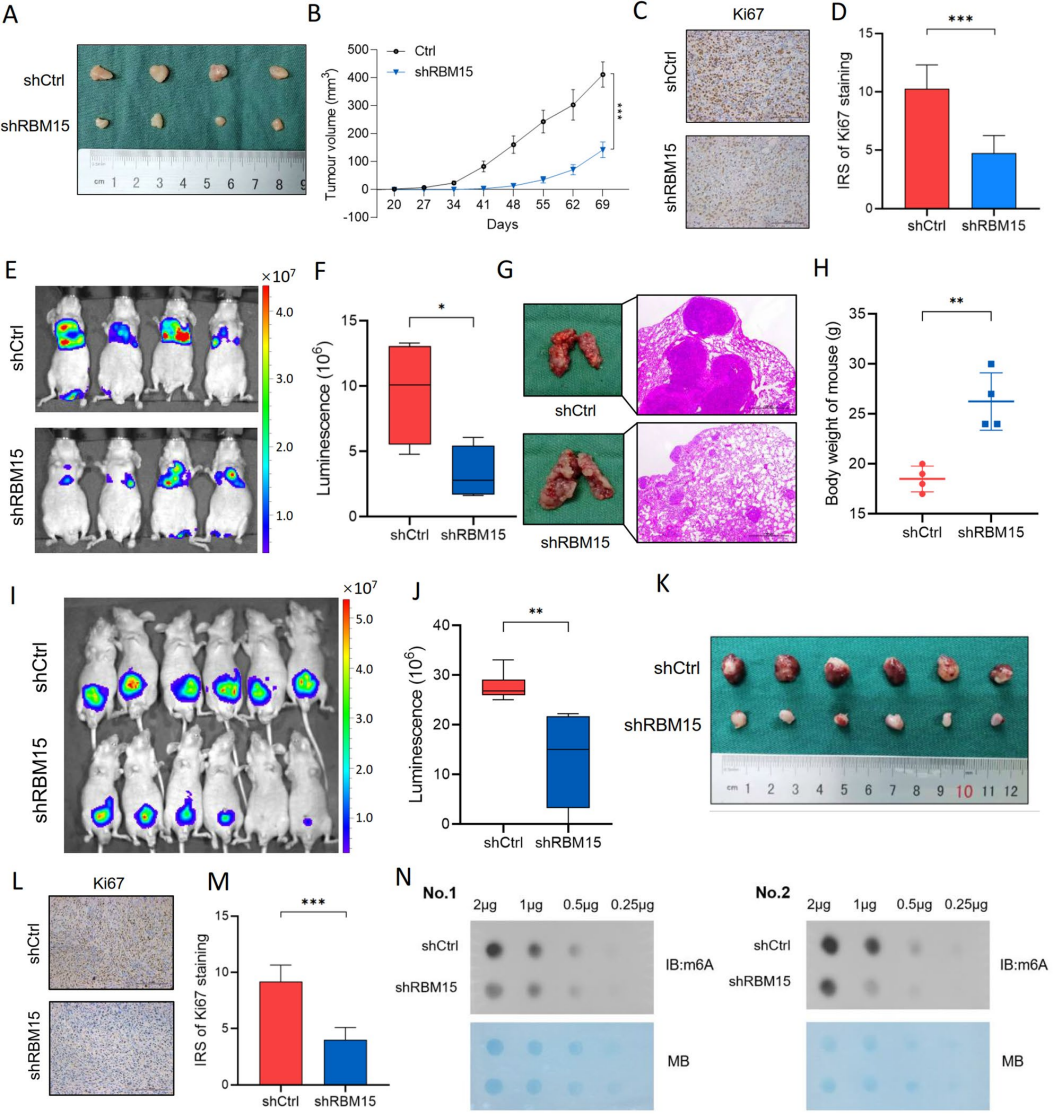
RBM15 promotes m6A-dependent BC growth and metastasis in vivo. **A-D**. Subcutaneous xenograft model. T24 cells with stable RBM15 knockdown (shRBM15) or control (shCtrl) cells were subcutaneously injected into BALB/c nude mice (*n* = 4 mice/group). **A** Representative images of excised tumors showing smaller tumors in the shRBM15 group. **B** Tumor growth curves displaying significantly suppressed tumor growth upon RBM15 knockdown. **C** Representative images of Ki67 IHC staining. Scale bar: 100×, 10× objective × 10× ocular. **D** Quantification of Ki67 staining showing reduced proliferation in shRBM15 tumors. **E-H**. Tail vein metastasis model. MB49-Luc+ cells transfected with shRBM15 or shCtrl were injected via the tail vein (*n* = 4 mice/group). **E** Representative bioluminescence images of lung metastases on Day 21. **F** The quantitative analysis of the metastatic burden based on the luminescent signal intensity revealed a dramatically reduced lung metastatic burden in the shRBM15 group. **G** Representative images of gross lung morphology and HE-stained sections. Scale bar: 40×, 10× objective × 4× ocular. **H** Weight measurements at the end of the experiment showing attenuated weight loss in the shRBM15 group. **I-N.** Orthotopic bladder xenograft model. MB49-Luc+ cells transfected with shRBM15 or shCtrl were implanted into the bladder wall (*n* = 6 mice/group). **I** In vivo bioluminescence imaging of orthotopic tumors. **J** Quantification of bioluminescence on day 30 showing a lower tumor burden in shRBM15 mice. **K** Representative images of ex vivo bladder tumors after the experimental endpoint. **L** Representative images of Ki67 IHC staining in orthotopic tumors. The scale bar is the same as in (**C**). **M** Quantification of Ki67 staining confirming reduced Ki67 staining in shRBM15 orthotopic tumors. **N** RNA dot blot analysis revealing decreased global m6A levels in shRBM15 orthotopic tumors. Methylene blue (MB) staining served as a loading control. **p < 0.01 and ***p < 0.001

We established a subcutaneous xenograft model using T24 cells with stable RBM15 knockdown to validate the oncogenic role of RBM15 in vivo. RBM15 depletion significantly suppressed tumor growth, as evidenced by the reduced tumor size and volume (Fig. 3A, B). IHC analysis confirmed a marked decrease in the number of Ki67-positive cells in the shRBM15 group (Fig. 3C, D).

We utilized the murine BC cell line MB49-Luc + to investigate the role of RBM15 in metastasis. RBM15 knockdown was confirmed (Fig. S2A), and consistent with our findings using human cells, RBM15 depletion impaired proliferation, migration, and invasion in vitro (Fig. S2B-D). In a tail vein injection model, RBM15-deficient cells generated dramatically fewer lung metastases with better-preserved alveolar structures (Fig. 3E-G). Notably, compared with control mice, mice in the shRBM15 group exhibited attenuated weight loss (Fig. 3H).

Finally, we established an orthotopic BC model. Bioluminescence imaging revealed a significantly lower tumor burden in the shRBM15 group (Fig. 3I, J), as confirmed by the observation of smaller tumors at necropsy (Fig. 3K) and reduced Ki67 staining (Fig. 3L, M). Crucially, an RNA dot blot analysis of orthotopic tumors showed decreased global m6A levels upon RBM15 knockdown (Fig. 3N).

Collectively, these in vivo data from three distinct models provide compelling evidence that RBM15 promotes BC tumorigenesis and metastasis, at least in part, by maintaining a state of m6A hypermethylation.

### RBM15 directly binds to and promotes the m6A-dependent degradation of the ZO2 mRNA

**Fig. 4:**
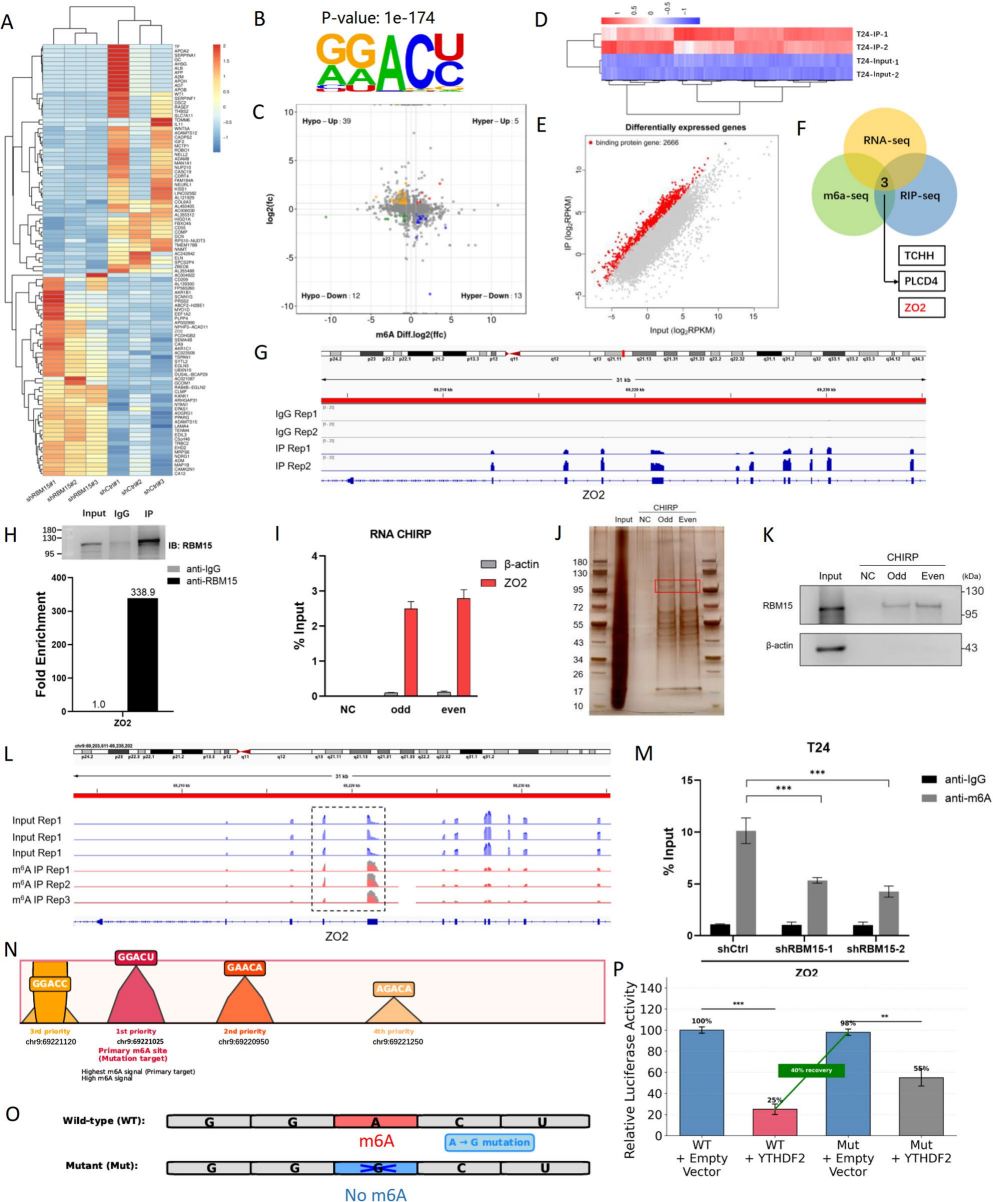
RBM15 directly binds to and mediates the m6A modification of the ZO2 mRNA to promote its degradation.** A** Heatmap of differentially expressed genes from the RNA-seq analysis of RBM15-knockdown and control T24 cells. **B** Analysis of the sequence motifs of RBM15-dependent m6A peaks showing the enrichment of the canonical RRACH motif. **C** Four-quadrant chart integrating RNA-seq and m6A-seq data, highlighting genes whose mRNA expression and m6A methylation changed significantly. **D** Heatmap displaying RBM15-bound transcripts identified through RIP-seq in T24 cells. **E** Scatter plot showing differentially expressed genes identified by RIP-seq that were directly bound by RBM15. **F** Venn diagram illustrating the overlap of differentially expressed genes (RNA-seq), differentially methylated genes (m6A-seq), and RBM15-bound transcripts (RIP-seq), identifying TCHH, PLCD4, and ZO2 as high-confidence targets. **G** IGV snapshot of RIP-seq data showing the enrichment of RBM15 binding peaks on the ZO2 transcript. **H** RIP-qPCR analysis confirming the direct interaction between RBM15 and the ZO2 mRNA in T24 cells. **I** CHIRP assay using biotin-labeled probes targeting the ZO2 mRNA, which successfully pulled down the ZO2 mRNA. **J** Silver staining of proteins that coprecipitated with the ZO2 mRNA, with the arrow indicating a band corresponding to the molecular weight of RBM15. **K** WB confirming the identity of the coprecipitated protein as RBM15. **L** IGV snapshot of m6A-seq data showing a significant RBM15-dependent m6A peak in the 3’UTR of the ZO2 transcript. **M** m6A-IP-qPCR analysis showing reduced m6A levels on the ZO2 mRNA upon RBM15 knockdown in T24 cells. **N** Schematic of the ZO2 3’UTR showing the location of the primary m6A site (GGACU). **O** Schematic of the luciferase reporter constructs containing the wild-type (WT) or mutant (Mut) ZO2 3’ UTR. **P** Luciferase reporter assay showing that the YTHDF2-mediated suppression of reporter activity was reversed by the A-to-G mutation at the m6A site. ***P < 0.001

We elucidated the molecular mechanism by which RBM15 performs its oncogenic functions by performing a multiomics analysis to identify its direct downstream targets. RNA sequencing (RNA-seq) of T24 cells following RBM15 knockdown revealed 164 upregulated genes and 176 downregulated genes (Fig. 4A; Fig. S3A). Concurrently, m6A sequencing (m6A-seq) revealed that RBM15-dependent m6A peaks were predominantly enriched with the canonical RRACH motif (Fig. 4B) and were primarily located in the 3’ UTR and exon regions (Fig. S3B), with 5,806 differentially abundant m6A peaks identified across 4,501 genes. An integrated analysis of RNA-seq and m6A-seq data revealed that upon RBM15 knockdown, 13 genes, including TCHH, PLCD4, and ZO2, exhibited both decreased m6A methylation and increased mRNA expression (Fig. 4C). RNA immunoprecipitation sequencing (RIP-seq) was conducted to further pinpoint the direct targets, and 2,666 genes whose transcripts were directly bound by the RBM15 protein were identified (Fig. 4D and E; Fig. S3C, S3D). Cross-referencing these three datasets yielded three high-confidence candidates: TCHH, PLCD4, and ZO2 (Fig. 4F). Zona Occludens 2 (ZO2), a key component of tight junctions and a regulator of cell signaling and proliferation, was selected as the primary candidate for further investigation because of its established role in cancer metastasis and its association with the epithelial‒mesenchymal transition (EMT) pathway [21].

We employed multiple orthogonal approaches to validate the direct interaction between RBM15 and the ZO2 mRNA. Visualization of the RIP-seq data using the Integrative Genomics Viewer (IGV) showed the significant enrichment of RBM15 binding peaks on the ZO2 transcript (Fig. 4G). This binding was biochemically confirmed by RIP‒qPCR, which demonstrated a robust and specific interaction between the endogenous RBM15 protein and ZO2 mRNA in both T24 and 5637 cells (Fig. 4H; Fig. S3E). We performed a CHIRP assay using biotin-labeled probes targeting the ZO2 mRNA to confirm this interaction in situ. The ZO2 mRNA was successfully pulled down in the CHIRP assay (Fig. 4I) and a distinct protein band at approximately 95–130 kDa was coprecipitated (Fig. 4J), which was subsequently identified as RBM15 by WB (Fig. 4K), thus confirming the physical association within the cellular context.

Next, we investigated whether RBM15 regulates the m6A modification of the ZO2 mRNA. The visualization of our m6A-seq data revealed a significant RBM15-dependent m6A peak within the 3’UTR of the ZO2 transcript (Fig. 4L). This finding was validated by m6A-IP‒qPCR, which showed a marked reduction in the level of the m6A modification of the ZO2 mRNA upon RBM15 knockdown in both T24 and 5637 cells (Fig. 4M; Fig. S3F). Bioinformatic analysis of the primary m6A peak (chr9:69220924–69221434) indicated four consensus DRACH motifs, with the GGACU sequence at chr9:69221025 showing the strongest and most consistent enrichment signal, identifying it as the primary m6A site (Fig. 4N; Table S14). The m6A modification typically promotes mRNA degradation through recognition by YTH domain-containing reader proteins, particularly YTHDF2, which recruits the CCR4–NOT deadenylase complex to accelerate mRNA decay [22]. We determined whether the identified m6A site on ZO2 mRNA is functional and mediates YTHDF2-dependent degradation by constructing luciferase reporter plasmids containing either the wild-type (WT) ZO2 3’UTR or a mutant version with an A-to-G substitution at the identified m6A site (Fig. 4O). Cotransfection with the YTHDF2 overexpression plasmid significantly suppressed WT reporter activity to approximately 25% of the control level. Critically, the A-to-G mutation, which abolishes the m6A modification at this site, partially rescued luciferase activity to 55%, representing a 40% recovery compared with that of the WT reporter (Fig. 4P; Table S15). These results demonstrate that the identified m6A site is functional and mediates the YTHDF2-dependent degradation of the ZO2 mRNA.

### RBM15 suppresses ZO2 expression to promote the EMT and regulates the nuclear translocation of ZO2 to control Snail expression

**Fig. 5:**
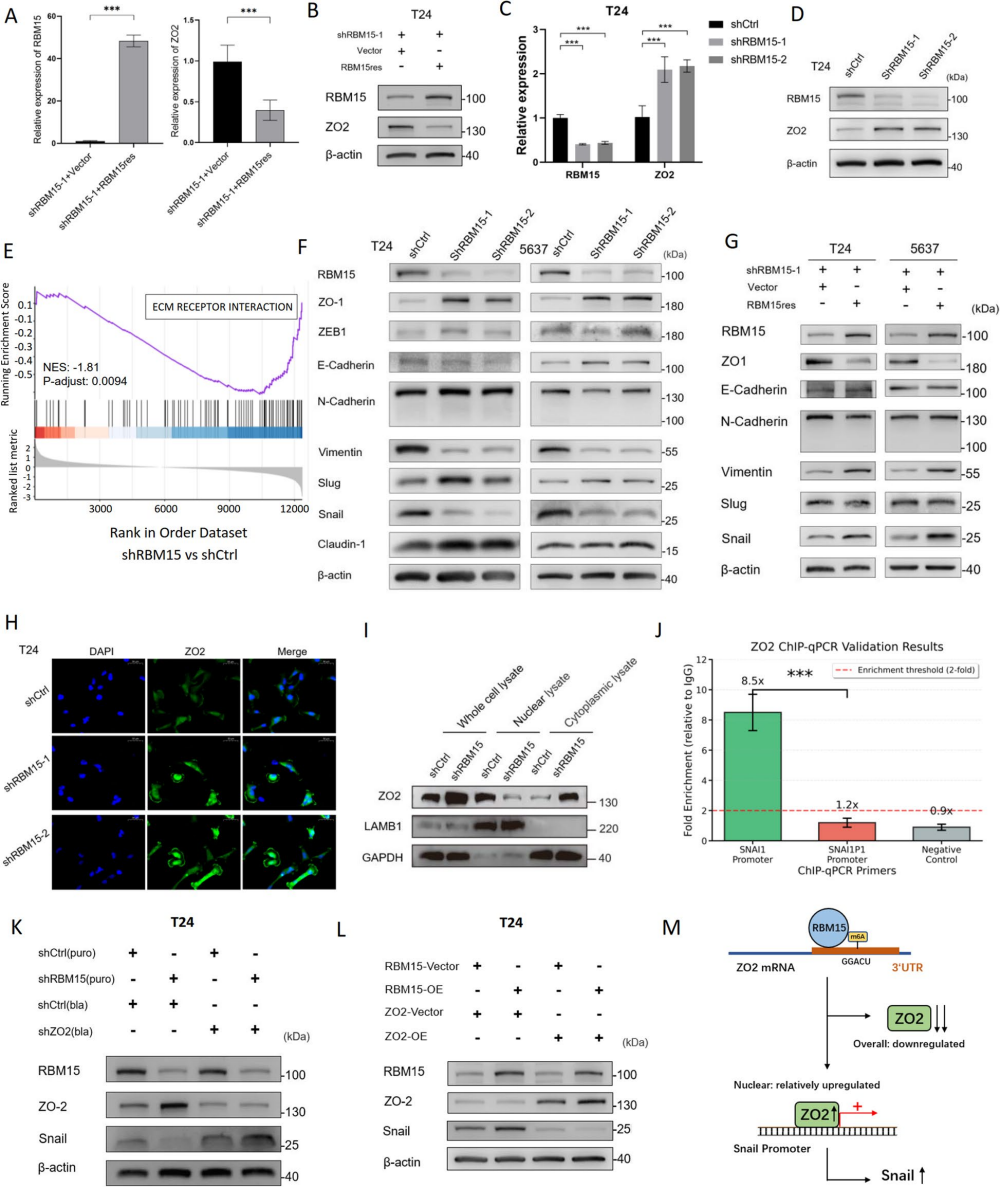
RBM15 drives the EMT by regulating the expression and nuclear localization of ZO2.** A**,** B** qPCR (**A**) and WB (**B**) analyses of RBM15 and ZO2 expression in T24 cells following RBM15 knockdown by shRNA. **C**,** D** Rescue of ZO2 expression was assessed by qPCR (**C**) and WB (**D**) in RBM15-knockdown T24 cells re-expressing RBM15 (shRBM15 + Vector vs. shRBM15 + RBM15res). **E** Gene set enrichment analysis (GSEA) plots showing the significant negative enrichment of pathway related to the extracellular matrix receptor interaction in RBM15-knockdown cells. **F** WB analysis of EMT-related markers in T24 and 5637 cells after RBM15 knockdown. **G** WB analysis of EMT markers in RBM15-knockdown T24 and 5637 cells following the re-expression of RBM15, indicating that Vimentin and Snail are the primary EMT-related proteins regulated by RBM15. **H** IF staining for ZO2 (green) in T24 cells after RBM15 knockdown. Nuclei were counterstained with DAPI (blue). **I** Nuclear and cytoplasmic fractions from RBM15-knockdown cells were subjected to WB to detect ZO2 protein levels. LAMB1 and GAPDH served as nuclear and cytoplasmic loading controls, respectively. **J** ChIP-qPCR validation of ZO2 enrichment at the SNAI1 promoter. IgG was used as a negative control, and a known nonbinding region served as a negative control region. **K** WB analysis of RBM15, ZO2, and Snail protein expression in T24 cells with single or double knockdown of RBM15 and/or ZO2. **L** WB analysis of RBM15, ZO2, and Snail protein expression in T24 cells with single or dual overexpression of RBM15 and/or ZO2. **M** Schematic model illustrating the proposed mechanism. The RBM15-mediated m6A modification promotes ZO2 mRNA degradation. Despite the global reduction in ZO2 levels, nuclear accumulation of ZO2 is paradoxically increased, and ZO2 then binds to the Snail promoter, leading to increased Snail expression and facilitating the epithelial-mesenchymal transition. ***p < 0.001

We first examined ZO2 expression following the modulation of RBM15 levels in BC cells to validate the potential regulation of ZO2 by RBM15, as suggested by our RNA sequencing data. As anticipated, stable knockdown of RBM15 in T24 and 5637 cells resulted in significant increases in both the mRNA and protein levels of ZO2 (Fig. 5A, B; Fig. S4A, B). We conducted rescue experiments in RBM15-knockdown cells to confirm the specificity of this negative regulatory relationship. Re-expression of RBM15 in these cells successfully reversed this effect, leading to a marked decrease in ZO2 expression (Fig. 5C, D; Fig. S4C, D). Furthermore, we corroborated these findings using a series of in vivo models. In the subcutaneous xenograft, lung metastasis, and orthotopic bladder transplantation models, tumor tissues from the RBM15-knockdown groups all exhibited significantly elevated ZO2 protein levels (Fig. S4E). Collectively, these in vitro and in vivo results establish RBM15 as a key negative regulator of ZO2 in BC.

Given the established link between ZO2 and the EMT, we performed a gene set enrichment analysis (GSEA) on our RNA-seq data. The analysis revealed a significant negative enrichment in gene sets related to extracellular matrix receptor interaction pathway in RBM15-knockdown cells (NES < -1.5), suggesting a potential role for RBM15 in regulating the EMT process (Fig. 5E). We explored this possibility by assessing the expression of key EMT markers. WB analysis showed that RBM15 knockdown led to marked downregulation of the mesenchymal markers Snail and Vimentin in both T24 and 5637 cells (Fig. 5F). This effect was specifically rescued by the re-expression of RBM15 (Fig. 5G), which strongly confirmed the presence of an RBM15/ZO2/EMT regulatory axis.

In addition to its function as a scaffold protein at tight junctions, ZO2 can translocate to the nucleus and act as a transcriptional coregulator [23]. Therefore, we next investigated whether RBM15 influences the subcellular localization of ZO2. IF staining revealed a dramatic increase in total ZO2 protein levels in both T24 and 5637 cells upon RBM15 knockdown, with the protein distributed in both the cytoplasm and the nucleus (Fig. 5H; Fig. S4F). We performed nuclear and cytoplasmic fractionation to more precisely quantify the relative distribution of ZO2 in different cellular compartments. Key findings emerged from the WB analysis: although the total ZO2 protein level was substantially increased following RBM15 knockdown, the amount of nuclear ZO2 was significantly decreased (Fig. 5I). Conversely, overexpression of RBM15 led to an increased relative abundance of nuclear ZO2 (Fig. S4G). These data collectively point to a central conclusion: RBM15 facilitates the nuclear translocation or retention of ZO2. When RBM15 is silenced, this nuclear transport is impaired. Consequently, although the cell produces a large amount of ZO2 protein because of the loss of m6A-mediated degradation, a smaller fraction of this protein successfully reaches the nucleus, leading to a functional decrease in nuclear ZO2 activity.

We performed a ChIP-seq analysis to identify the direct downstream targets of nuclear ZO2, which revealed ZO2 binding peaks in the promoter regions of several genes. Notably, a prominent peak was annotated to the promoter of SNAI1P1, a pseudogene of Snail (Fig. S4H). Considering the high sequence homology between the promoter regions of Snail and its pseudogene, we designed highly specific primers to distinguish between these sites in a validation ChIP‒qPCR experiment. The results unequivocally demonstrated that ZO2 is significantly enriched at the functional SNAI1 promoter but not at the SNAI1P1 promoter or a negative control region (Fig. 5J), providing conclusive evidence that ZO2 directly binds to the SNAI1 promoter. We examined Snail expression to confirm the functional consequences of this binding. Consistent with our hypothesis, RBM15 knockdown in T24 cells upregulated ZO2 expression but downregulated Snail expression, whereas the knockdown of ZO2 alone upregulated Snail expression. A double-knockdown experiment confirmed that ZO2 acts downstream of RBM15, as Snail expression remained high despite the loss of RBM15 when ZO2 was also silenced (Fig. 5K; Fig. S4I). Conversely, the overexpression of RBM15 downregulated ZO2 expression and upregulated Snail expression, an effect that was reversed by the co-overexpression of ZO2 (Fig. 5L; Fig. S4J). This intricate regulatory cascade demonstrates that by controlling the m6A modification and nuclear availability of ZO2, RBM15 ultimately modulates Snail expression to drive the EMT and the malignant phenotype of BC (Fig. 5M).

### RBM15 drives oncogenic phenotypes in BC via ZO2-dependent pathways

**Fig. 6:**
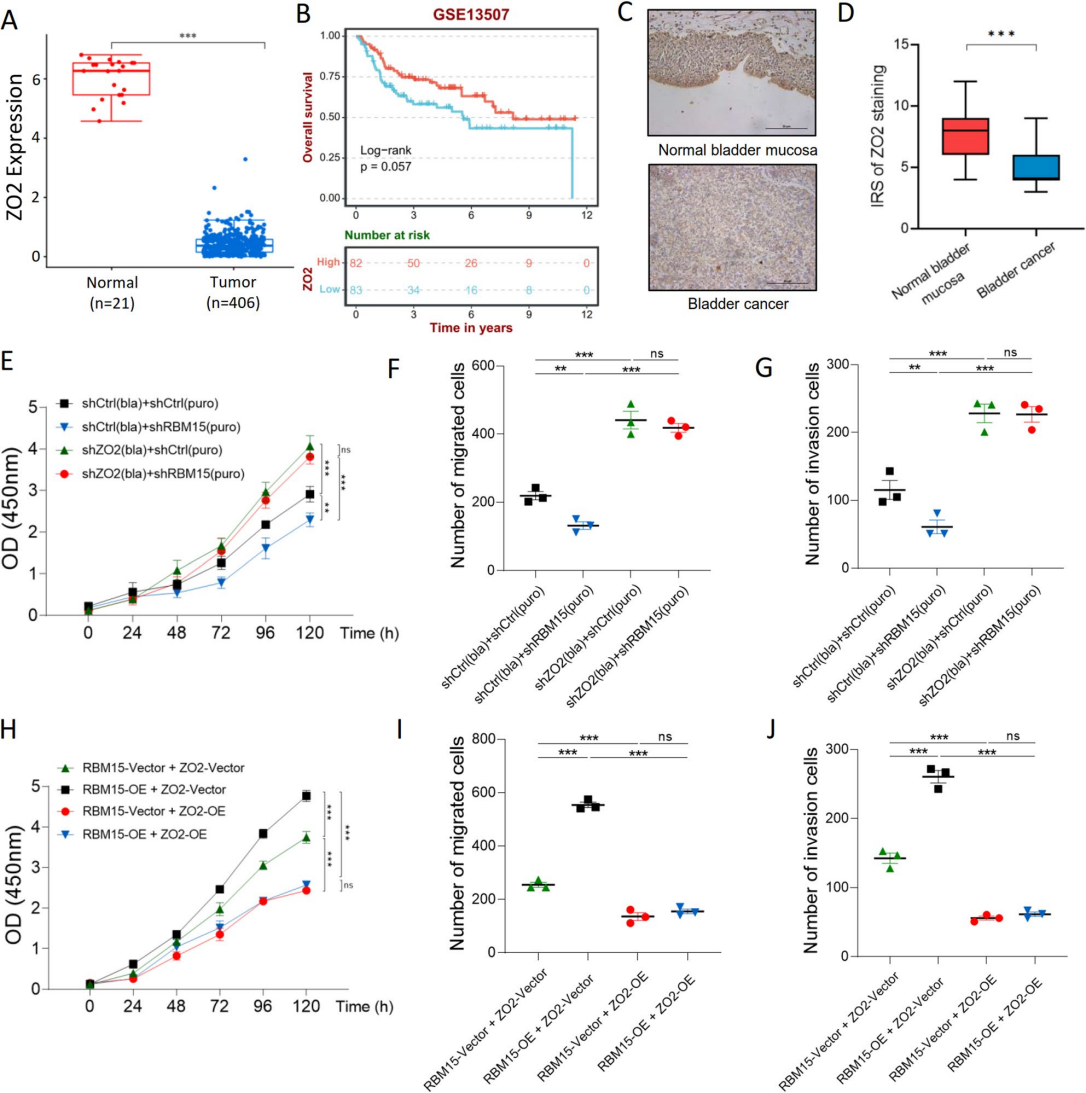
RBM15 promotes BC progression by suppressing the downstream tumor suppressor ZO2.** A** ZO2 expression is significantly downregulated in BC tissues (*n* = 406) compared with normal tissues (*n* = 21) from TCGA and GTEx datasets. **B** A Kaplan-Meier analysis of the GSE13507 dataset showed that low ZO2 expression correlates with shorter overall survival of BC patients. **C**,** D** Representative images of IHC staining (**C**) and the corresponding quantitative analysis (**D**) demonstrate reduced ZO2 protein levels in BC tissues compared with those in paired adjacent normal bladder mucosa. **E-G** The inhibitory effects of RBM15 knockdown on cell proliferation (**E**), migration (**F**), and invasion (**G**) in T24 cells were reversed by the simultaneous knockdown of ZO2. **H-J** The protumorigenic effects of RBM15 overexpression on cell proliferation (**H**), migration (**I**), and invasion (**J**) in T24 cells were significantly attenuated by the co-overexpression of ZO2. **p < 0.01, ***p < 0.001, and ns, *p* > 0.05

We first analyzed ZO2 expression in publicly available datasets and clinical samples to determine its clinical relevance and biological function in BC. The role of ZO2 in BC is poorly defined. An analysis of the data from TCGA and GTEx databases revealed that ZO2 expression was significantly lower in BC tumor tissues than in normal tissues (Fig. 6A). Furthermore, an analysis of the GSE13507 dataset indicated that patients with low ZO2 expression experienced significantly shorter overall survival (Fig. 6B). We validated these findings in our own cohort of paired clinical samples, where IHC confirmed a marked decrease in ZO2 protein levels in BC tissues compared with those in adjacent normal bladder mucosa (Fig. 6C, D).

We performed loss-of-function studies to directly assess the function of ZO2. Efficient knockdown of ZO2 in both T24 and 5637 cells was confirmed by WB and qPCR (Fig. S5A, B). Consistent with its clinical correlation with longer survival, ZO2 knockdown significantly increased cell proliferation, migration, and invasion in both cell lines (Fig. S5C-H), establishing its tumor-suppressive function in BC.

Given that RBM15 negatively regulates ZO2, we next sought to determine whether ZO2 is a critical downstream effector in the RBM15-driven oncogenic pathway. As previously established, knockdown of the oncogene RBM15 impaired the proliferation, migration, and invasion of T24 cells. Conversely, the depletion of the tumor suppressor ZO2 enhanced these malignant phenotypes. Critically, in a dual-knockdown setting, the concurrent depletion of ZO2 completely reversed the antitumor effects of RBM15 loss, sustaining the hyperproliferative and invasive capacities of the cancer cells (Fig. 6E-G). This epistatic relationship was further validated in 5637 cells (Fig. S5I-K), providing compelling evidence that ZO2 acts as a crucial functional downstream effector of RBM15.

We conducted gain-of-function experiments to further solidify this hierarchical axis. The overexpression of RBM15 in T24 cells promoted their proliferation, migration, and invasion. However, this protumorigenic effect was significantly abrogated by the co-overexpression of ZO2 (Fig. 6H-J). Similar results were obtained in 5637 cells (Fig. S5L-N). Together, these results establish a clear causal relationship, positioning ZO2 as an essential, nonredundant tumor suppressor whose functional loss is a key event downstream of the oncogenic signaling of RBM15.

### RBM15 facilitates ZO2 mRNA degradation via the YTHDF2-dependent recognition of m6A modifications in BC

Having established that RBM15 downregulates ZO2 expression via the m6A modification, we next sought to identify the m6A reader protein responsible for executing this effect. Given the well-established role of YTHDF2 in promoting the degradation of m6A-modified mRNAs [24], we hypothesized that it might be the key effector mediating the decay of ZO2 transcripts. Bioinformatics analysis using the m6A2Target database predicted a direct interaction between YTHDF2 and the ZO2 mRNA (Fig. S6A). Consistent with its potential oncogenic role, YTHDF2 was significantly upregulated in BC tissues in TCGA dataset (Fig. S6B).

**Fig. 7:**
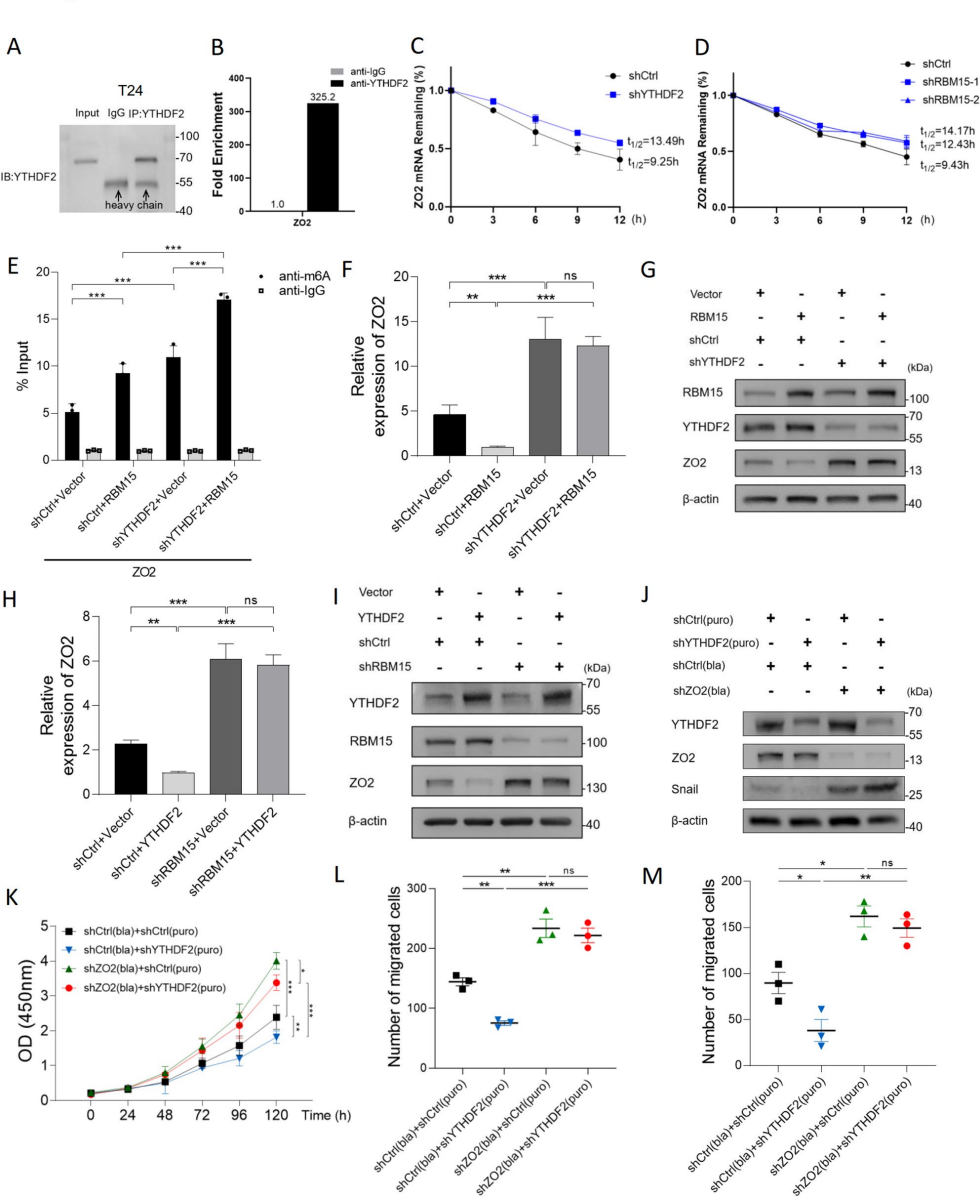
The RBM15-induced m6A modification of the ZO2 mRNA leads to its degradation by YTHDF2 in T24 cells.** A**,** B **RIP followed by WB (**A**) and qPCR (**B**) demonstrated a direct binding interaction between the YTHDF2 protein and ZO2 mRNA in T24 cells. **C**,** D** mRNA stability assays showed that knockdown of either YTHDF2 (**C**) or RBM15 (**D**) significantly increases the half-life of the ZO2 mRNA. **E** m6A-IP-qPCR analysis revealed that RBM15 overexpression increases the level of the m6A modification of ZO2 transcripts, an effect that is reversed by YTHDF2 knockdown. **F**,** G**. The suppression of ZO2 mRNA (**F**) and protein (**G**) expression by RBM15 overexpression depends on the presence of YTHDF2, as its knockdown rescues ZO2 expression. **H**,** I** The downregulation of ZO2 mRNA (**H**) and protein (**I**) expression induced by YTHDF2 overexpression is contingent on RBM15, as RBM15 knockdown reverses this effect. **J** The suppression of the expression of the mesenchymal marker Snail by YTHDF2 knockdown is reversed by the simultaneous depletion of ZO2. **K-M** The inhibitory effects of YTHDF2 knockdown on cell proliferation (**K**), migration (**L**), and invasion (**M**) are reversed by the coknockdown of ZO2. *p < 0.05, **p < 0.01, ***p < 0.001, and ns, *p* > 0.05

We performed RNA immunoprecipitation (RIP)–qPCR to experimentally validate this interaction, and the results confirmed the significant enrichment of the ZO2 mRNA in YTHDF2 immunoprecipitates from both T24 and 5637 cells (Fig. 7A, B; Fig. S7A, B). We then assessed the functional consequence of this binding on mRNA stability. Using actinomycin D to block new transcription, we observed that YTHDF2 knockdown markedly extended the half-life of the ZO2 mRNA in both cell lines (Fig. 7C; Fig. S7C). A similar increase in ZO2 mRNA stability was observed following RBM15 depletion (Fig. 7D; Fig. S7D), strongly suggesting that both RBM15 and YTHDF2 are involved in the same m6A-dependent mRNA destabilization pathway.

We conducted a series of rescue experiments to elucidate the hierarchical relationship within the RBM15–YTHDF2–ZO2 axis. First, we confirmed that RBM15 overexpression led to a significant increase in the level of the m6A modification of ZO2 transcripts (Fig. 7E). As expected, RBM15 overexpression suppressed ZO2 mRNA and protein expression; however, this suppression was completely abrogated upon the simultaneous knockdown of YTHDF2 (Fig. 7F, G). Conversely, while YTHDF2 overexpression alone was sufficient to reduce ZO2 expression, this effect was abolished when RBM15 was knocked down (Fig. 7H, I). These results were consistently replicated in 5637 cells (Fig. S7E-I), providing conclusive evidence that the YTHDF2-mediated degradation of the ZO2 mRNA is contingent upon the m6A marks installed by RBM15.

Finally, we investigated the functional implications of this axis. YTHDF2 knockdown phenocopied the effects of RBM15 knockdown, leading to the suppression of the expression of the mesenchymal marker Snail. Crucially, the simultaneous depletion of ZO2 reversed this change, restoring the mesenchymal phenotype (Fig. 7J). Similarly, YTHDF2 silencing inhibited cell proliferation, migration, and invasion, but these tumor-suppressive effects were fully reversed by the coknockdown of ZO2 (Fig. 7K-M). The results of these functional rescue experiments, which were also validated in 5637 cells (Fig. S7J-M), confirmed that YTHDF2 affects BC progression primarily through the regulation of its critical downstream target, ZO2.

### YTHDF2 requires m6A-binding activity to degrade the ZO2 mRNA and drive BC malignancy

**Fig. 8:**
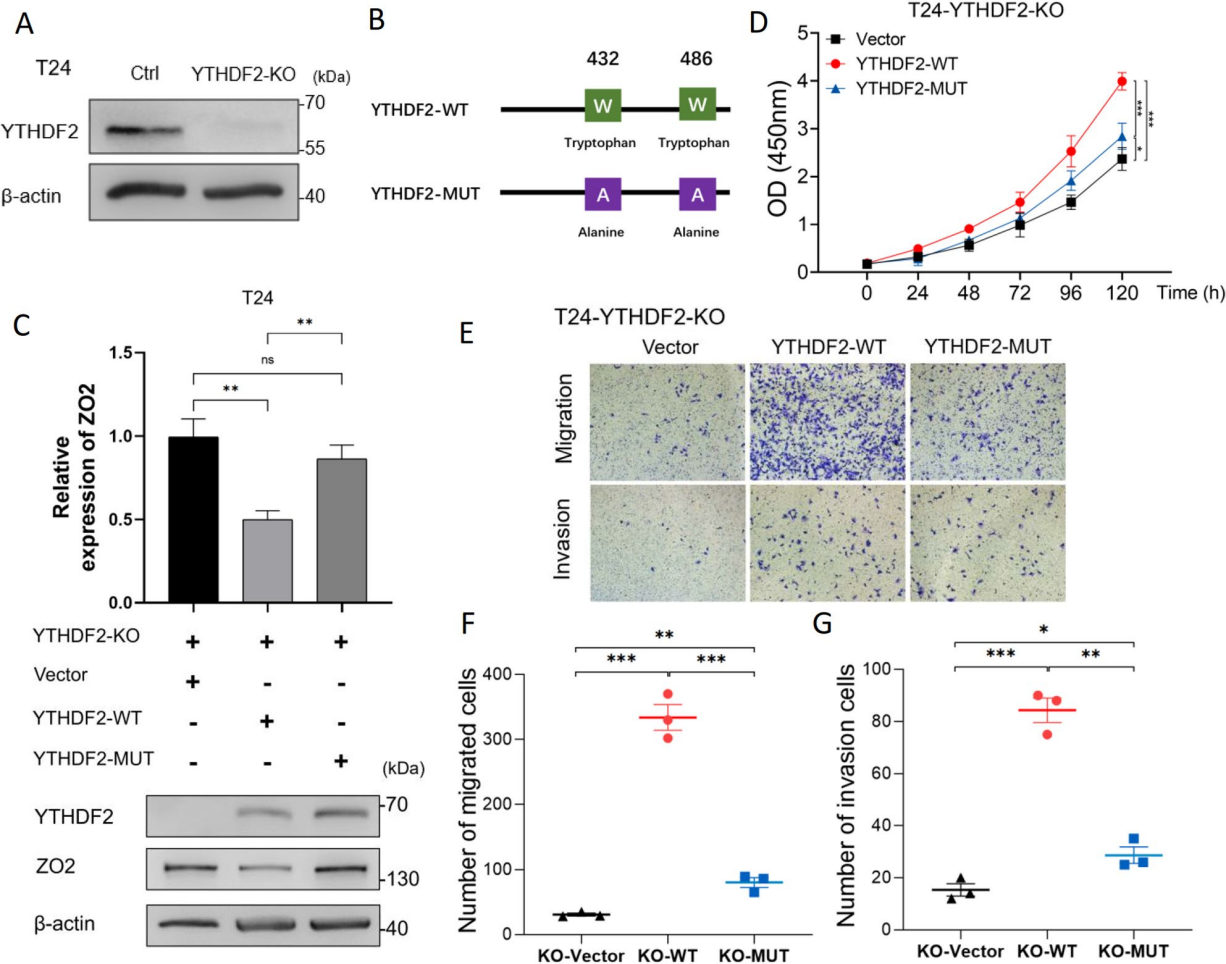
The m6A-binding activity of YTHDF2 is essential for its oncogenic function in BC cells. **A** WB confirming YTHDF2 knockout in T24 cells. **B** Schematic of YTHDF2-WT and the m6A-binding-deficient mutant (W432A/W486A). The mutant harbors W432A and W486A substitutions in the conserved m6A recognition pocket. **C** qPCR analysis (upper panel) and WB analysis (lower panel) of ZO2 expression in YTHDF2-KO T24 cells reconstituted with empty vector (Vector), YTHDF2-WT, or YTHDF2-MUT. YTHDF2-WT suppressed ZO2; YTHDF2-MUT failed to reduce ZO2 expression. **D** CCK-8 assay measuring cell proliferation in YTHDF2-KO T24 cells reconstituted with the indicated constructs over a 120-hour time course. YTHDF2-WT fully rescued proliferation, whereas YTHDF2-MUT partially rescued proliferation. **E** Representative images of the Transwell migration (upper panel) and invasion (lower panel) assay. **F**,** G** Quantification of migrated (**F**) and invaded (**G**) cells from the Transwell assays. YTHDF2-WT restored migration/invasion, whereas YTHDF2-MUT resulted in ~ 20% of the effect of the WT protein. *p < 0.05, **p < 0.01, and ***p < 0.001

We generated YTHDF2 knockout (KO) T24 and 5637 cells to assess the m6A-binding pocket of YTHDF2 (Fig. 8A; Fig. S8A). Functional assays showed that YTHDF2 depletion significantly suppressed cell proliferation, migration, and invasion in both cell lines (Fig. S8B-I), confirming the protumorigenic role of YTHDF2 in BC.

We performed rescue experiments in YTHDF2-KO T24 cells to further elucidate the underlying molecular mechanism. Reconstitution with wild-type YTHDF2 (YTHDF2-WT) markedly suppressed ZO2 expression at both the mRNA and protein levels and fully restored the malignant phenotypes, including proliferation, migration, and invasion, that were impaired by YTHDF2 knockout. In striking contrast, the m6A-binding-deficient mutant (YTHDF2-MUT, W432A/W486A) (Fig. 8B), which harbors point mutations in the conserved m6A recognition pocket [25–27], failed to effectively suppress ZO2 expression and only weakly rescued the malignant phenotype, exhibiting approximately 20% of the effect of the wild-type protein on migration and invasion (Fig. 8C-G).

Consistent findings were observed in the 5637 cells (Fig. S9A-E). The residual activity of YTHDF2-MUT suggests the existence of minor m6A-independent functions. However, these data collectively demonstrate that the m6A-binding activity of YTHDF2 is indispensable for its primary oncogenic functions.

Taken together, these results conclusively indicate that YTHDF2 requires its m6A-binding domain to recognize and degrade the ZO2 mRNA, thereby promoting BC malignancy.

### RBM15 drives m6A hypermethylation in BC by binding to the METTL3-WTAP-METTL14 methyltransferase complex

The mechanism by which RBM15 increases m6A levels in BC is unclear. Given the central catalytic role of METTL3 in the methyltransferase 3-Wilms’ tumor 1-associating protein–methyltransferase 14 (METTL3-WTAP-METTL14) methyltransferase complex, we hypothesized that RBM15 acts through this complex to promote m6A deposition and tumor progression.

**Fig. 9:**
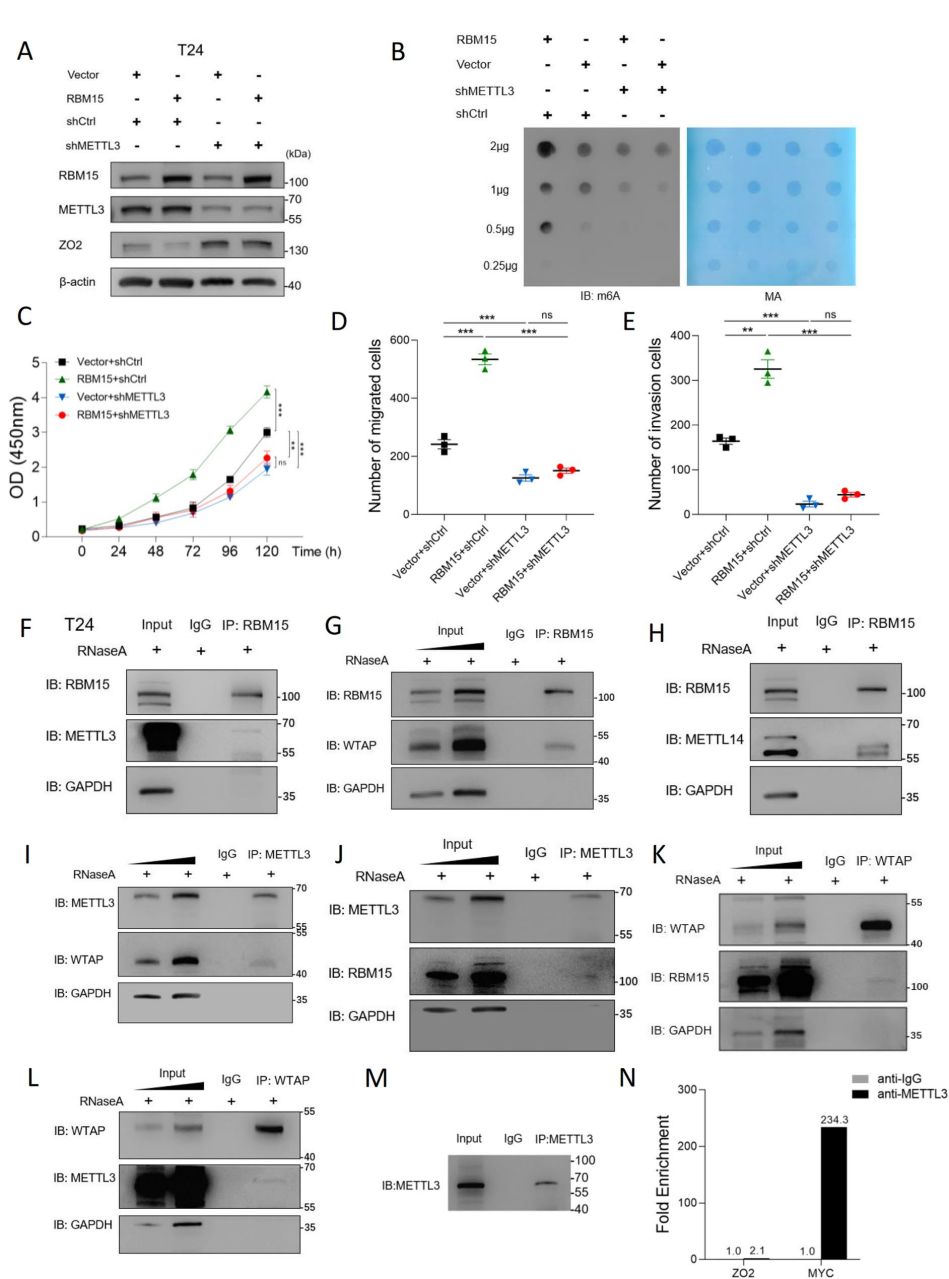
RBM15 drives m6A hypermethylation and oncogenesis via collaboration with the METTL3–WTAP–METTL14 methyltransferase complex. **A**,** B** METTL3 is required for RBM15-mediated ZO2 repression and m6A modification in T24 cells. **A** METTL3 knockdown abrogates the ability of RBM15 overexpression to downregulate ZO2 mRNA and protein expression. **B** Global m6A levels, as determined by RNA dot blot assays, reveal that METTL3 depletion prevents RBM15-driven m6A hypermethylation. MB, methylene blue. **C–E** The oncogenic functions of RBM15 depend on METTL3 in T24 cells. **C** RBM15 overexpression failed to rescue the cell proliferation impaired by METTL3 knockdown. Migration (**D**) and invasion (**E**) assays revealed that RBM15 cannot restore migratory or invasive capabilities when METTL3 is depleted. **F-L** RBM15 interacts with the METTL3–WTAP–METTL14 core complex in T24 cells. **F**-**H** Co-IP using an anti-RBM15 antibody pulled down METTL3 (**F**), METTL14 (**G**), and WTAP (**H**). **I**–**L** Reciprocal Co-IP: the METTL3 antibody coprecipitates WTAP (**I**) and RBM15 (**J**); the WTAP antibody coprecipitates RBM15 (**K**) and METTL3 (**L**). **M**,** N** METTL3 does not directly bind to the ZO2 mRNA. RIP–qPCR in T24 cells revealed no enrichment of ZO2 transcripts among the METTL3 IP products, in contrast to the positive control MYC. **p < 0.01, ***p < 0.001, and ns, *p* > 0.05

In both T24 and 5637 cells, RBM15 overexpression reduced ZO2 expression, whereas METTL3 knockdown upregulated ZO2 expression. Notably, METTL3 loss abolished the effect of RBM15: ZO2 levels and global m6A levels remained unchanged despite RBM15 overexpression (Fig. 9A and B; Fig. S10A and B), indicating that RBM15 requires METTL3 to install m6A. Functionally, RBM15 could not rescue the impaired proliferation, migration, or invasion caused by METTL3 depletion (Fig. 9C-E; Fig. S10C-E).

Coimmunoprecipitation (Co-IP) confirmed that RBM15 interacts with METTL3, WTAP, and METTL14 in T24 cells (Fig. 9F, G, and H), with reciprocal Co-IPs showing the same results (Fig. 9I–L). Similar interactions were observed in the 5637 cells (Fig. S10F-K). Despite reports that METTL3 can bind certain mRNAs directly (e.g., MYC and AFF4) [10], RIP-qPCR showed that METTL3 does not bind the ZO2 mRNA directly (Fig. 9M and N; Fig. S10L and M). These findings suggest that RBM15 is the critical adaptor protein responsible for recruiting the methyltransferase complex to the ZO2 mRNA. 

### STM2457 inhibits BC progression via the METTL3/RBM15/ZO2-m6A axis in preclinical models

**Fig. 10:**
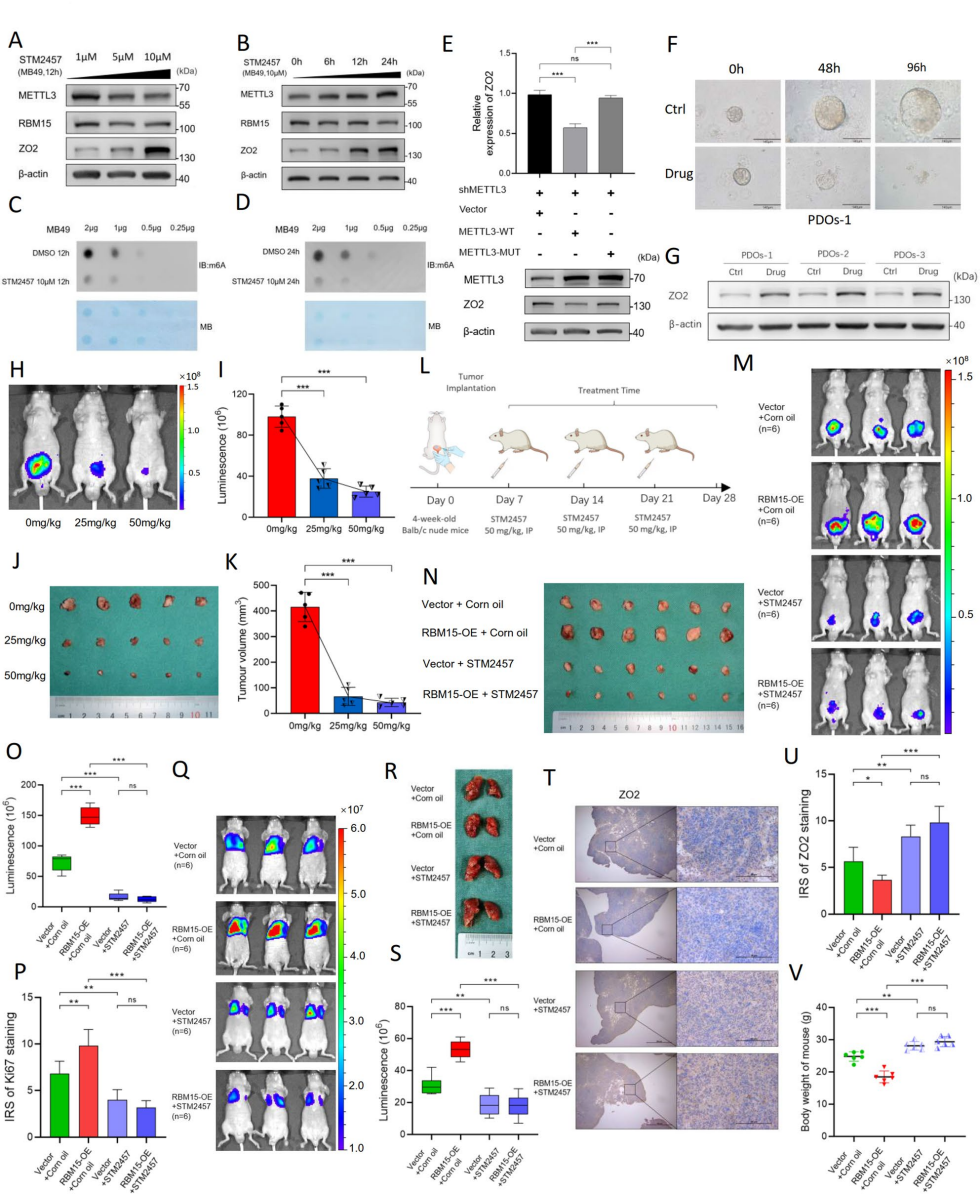
STM2457 inhibits BC progression via the METTL3/RBM15/ZO2-m6A axis in preclinical models. **A**,** B** WBs showing the dose-dependent (A: 1–10 µM, 12 h) and time-dependent (B: 10 µM, 0–24 h) upregulation of ZO2 expression by STM2457 in MB49 cells; METTL3 and RBM15 levels remained unchanged. **C**,** D** Dot blot analysis of global m6A levels in MB49 cells treated with STM2457 (10 µM) for 12 h (**C**) or 24 h (**D**). Methylene blue (MB) staining was used as a loading control. STM2457 reduces m6A levels. **E** qPCR (upper panel) and WB (lower panel) results showing ZO2 levels in METTL3-knockdown cells reconstituted with vector, METTL3-WT, or METTL3-D395A (catalytic-dead mutant). Only METTL3-WT suppressed ZO2 expression. **F** Representative images of PDO-1 treated with STM2457 (10 µM) at 0, 48, and 96 h. STM2457 inhibits organoid growth. **G** WB analysis of ZO2 expression in three independent PDOs (PDO-1, -2, and − 3) treated with STM2457. Drug treatment increases ZO2 expression. **H-K** STM2457 inhibits orthotopic bladder tumor growth (*n* = 5 mice per group). **H** Representative bioluminescence images of orthotopic bladder tumors in mice treated with vehicle (0 mg/kg), 25 mg/kg, or 50 mg/kg STM2457. **I** Quantification of bioluminescence showing dose-dependent tumor suppression by STM2457. **J** Representative images of excised BCs after the dose-dependent experimental endpoint. **K** Quantification of the tumor volume confirming a dose-dependent reduction. **L-P** STM2457 overcomes RBM15-driven tumor aggressiveness (*n* = 6 mice in each group). **L** Schematic of the orthotopic model with RBM15 overexpression and an STM2457 treatment timeline. **M** Representative bioluminescence images showing that STM2457 attenuates tumor growth in both the vector and RBM15-OE groups. **N** Representative images of excised bladder tissues from each group. **O** Bioluminescence quantification at the endpoint. STM2457 significantly reduces the tumor burden regardless of the RBM15 status. **P** Quantification of the Ki67 IRS showing reduced proliferation in response to STM2457 treatment. **Q-V** STM2457 blocks RBM15-mediated lung metastasis (*n* = 6 mice in each group). **Q** Representative bioluminescence images of the lung metastasis model. **R** Representative images of excised lungs showing metastatic nodules. **S** Quantification of lung metastatic burden. RBM15-OE increases metastasis, whereas STM2457 significantly attenuates the metastatic burden. **T** Representative images of ZO2 IHC staining in lung metastases. **U** IRS quantification of ZO2 expression. STM2457 restores ZO2 expression in metastatic lesions. **V** Body weight measurements showing improved weight maintenance in the STM2457-treated groups. **p < 0.01, ***p < 0.001, and ns, *p* > 0.05

The METTL3 inhibitor STM2457, which has demonstrated efficacy against leukemia and neuroblastoma [28], has shown promising therapeutic potential in BC. In MB49 cells, STM2457 treatment upregulated ZO2 expression in both a dose-dependent (1–10 µM, 12 h) and time-dependent (10 µM, 0–24 h) manner without altering METTL3 or RBM15 protein levels (Fig. 10A and B). Concurrently, a dot blot analysis revealed that STM2457 significantly reduced global m6A levels (Fig. 10C and D), confirming the on-target inhibition of METTL3 catalytic activity.

We genetically validated that METTL3 catalytic activity is essential for ZO2 regulation by performing rescue experiments using wild-type METTL3 (METTL3-WT) and a catalytically dead mutant (METTL3-D395A) in METTL3-knockdown cells [29, 30]. Only METTL3-WT restored the m6A modification of the ZO2 mRNA and suppressed ZO2 expression at both the mRNA and protein levels, whereas METTL3-D395A failed to do so (Fig. 10E and Fig. S11A). These results definitively demonstrate that METTL3 methyltransferase activity, rather than the protein itself, is required for RBM15-mediated ZO2 downregulation.

We performed drug sensitivity experiments to determine whether ZO2 upregulation mediates the anticancer effect of STM2457. ZO2 knockdown resulted in significant resistance to STM2457, with the IC50 values increasing approximately 5-fold compared with those of the controls (50.5 µM vs. 10.3 µM; Fig. S11B), indicating that ZO2 restoration is a primary contributor to the therapeutic efficacy of STM2457. Importantly, STM2457 exhibited greater than 10-fold selectivity for cancer cells versus non-malignant SV-HUC-1 urothelial cells (IC50: 11.6 µM and 7.6 µM for T24 and 5637 cells vs. 121.3 µM for SV-HUC-1 cells; Fig. S11C), suggesting a favorable therapeutic window for potential clinical application.

We established patient-derived organoids (PDOs) from three newly diagnosed patients with BC to evaluate the clinical relevance of our findings. STM2457 treatment (10 µM) significantly inhibited organoid growth in a time-dependent manner across all three PDO models (Fig. 10F and Fig. S11D), accompanied by marked upregulation of ZO2 expression (Fig. 10G). The quantitative analysis confirmed that STM2457-treated organoids showed significantly reduced growth compared with vehicle-treated control organoids (Fig. S11E), validating our proposed mechanism in clinically relevant models.

We next evaluated the efficacy of STM2457 in orthotopic xenograft models. Daily intraperitoneal administration of STM2457 (25–50 mg/kg) resulted in dose-dependent tumor suppression, as evidenced by reduced bioluminescence signals (Fig. 10H and I), decreased tumor size and volume (Fig. 10J and K), diminished Ki67 expression indicating reduced proliferation (Fig. S12A), and significantly restored ZO2 expression (Fig. S12B). Importantly, a comprehensive toxicity assessment revealed no observable adverse effects, as the histological analysis of lung, liver, and kidney tissues revealed a normal architecture (Fig. S12C), and serum levels of hepatic enzymes (ALT and AST) and renal function markers (creatinine and BUN) remained within normal ranges (Fig. S12D-G).

We investigated whether STM2457 could overcome RBM15-driven tumor progression by establishing orthotopic tumors using RBM15-overexpressing MB49 cells (Fig. S12H). As expected, compared with the vector control, RBM15 overexpression promoted tumor growth. Notably, compared with the control treatment, treatment with STM2457 (50 mg/kg) significantly attenuated tumor growth in RBM15-overexpressing tumors, with an efficacy comparable to that observed in control tumors (Fig. 10L-O; Fig. S12J). An ex vivo immunohistochemical analysis confirmed that STM2457 reduced cellular proliferation (Ki67 staining) and restored ZO2 expression in both vector- and RBM15-overexpressing tumors (Fig. 10P; Fig. S12I and K), demonstrating that pharmacological METTL3 inhibition can effectively reverse the oncogenic effects of RBM15 overexpression.

Finally, we assessed the efficacy of STM2457 in an experimental lung metastasis model. Compared with the injection of vector-expressing control cells, the tail vein injection of RBM15-overexpressing MB49 cells resulted in significantly increased lung colonization, confirming the prometastatic role of RBM15. Strikingly, compared with the vehicle treatment, STM2457 treatment dramatically attenuated the metastatic burden in the RBM15-overexpressing groups, reducing the number of lung metastatic nodules to levels comparable to or lower than those in the vehicle-treated vector control group (Fig. 10Q-S). The immunohistochemical analysis of lung metastatic lesions revealed that STM2457 treatment significantly restored ZO2 expression (Fig. 10T and U), consistent with the proposed mechanism of action. Furthermore, compared with vehicle-treated mice, mice in the STM2457-treated groups maintained significantly better body weight (Fig. 10V), indicating an improved overall health status and reduced tumor-associated cachexia.

Collectively, given that RBM15 overexpression occurs in approximately 70% of BC patients and is correlated with a poor prognosis, these preclinical data establish STM2457 as a promising therapeutic candidate for targeting aggressive, RBM15-high BC through the modulation of the METTL3/RBM15/ZO2 axis.

## Discussion

**Fig. 11:**
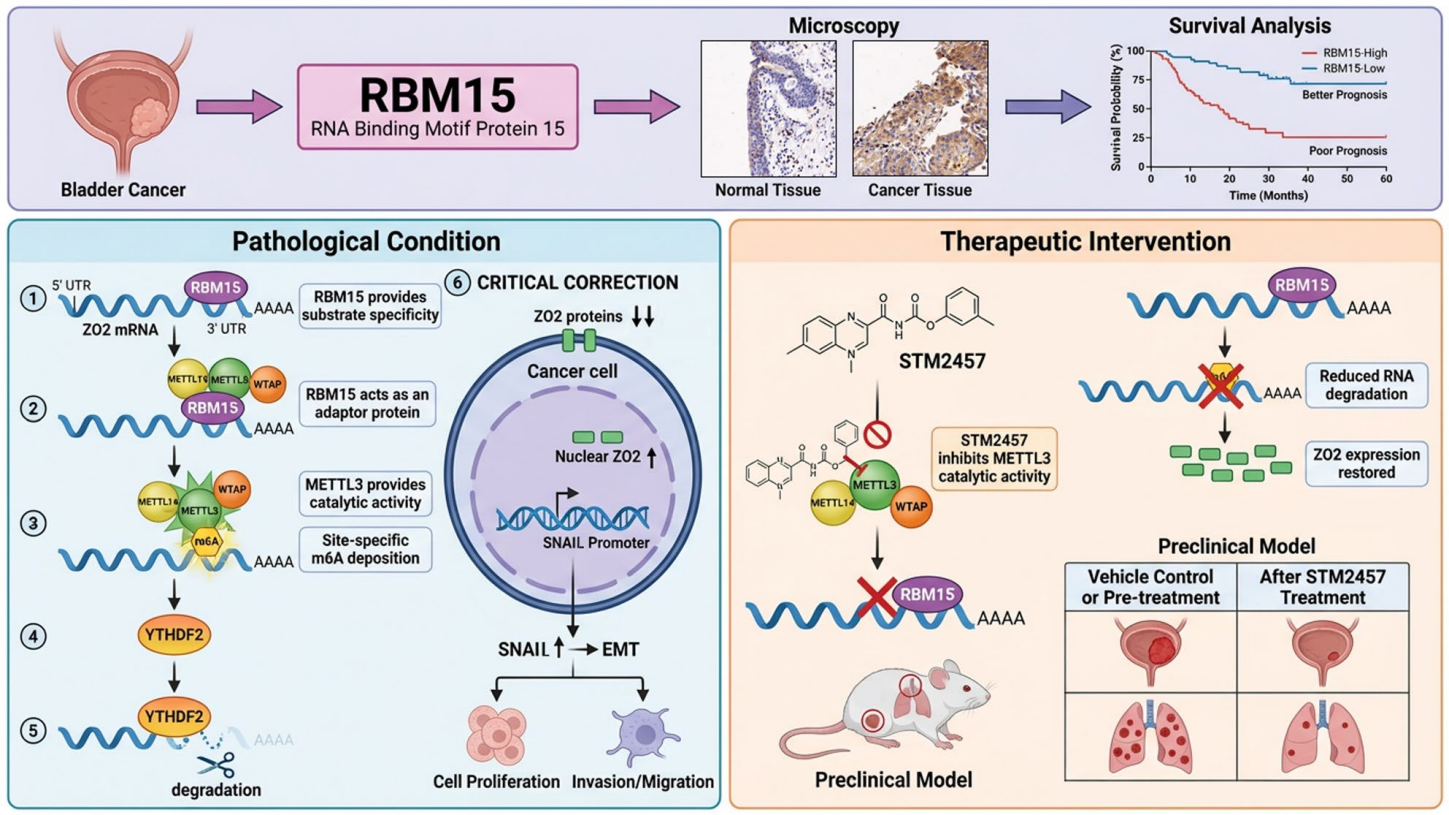
Graphical summary of the results of this study. RBM15 overexpression in BC drives a poor prognosis through the recruitment of the m6A methyltransferase complex METTL3-METTL14-WTAP to increase m6A modifications on the 3'UTR of the ZO2 mRNA. YTHDF2 recognizes the m6A-modified ZO2 mRNA, triggering mRNA decay. Although total ZO2 levels decrease, its preferential nuclear localization increases, promoting Snail expression and EMT progression. The METTL3 inhibitor STM2457 blocks this axis, suppressing tumor growth and metastasis in preclinical models

In this study, RBM15 was established as a critical m6A regulator that drives BC progression through a novel degradation mechanism targeting ZO2. While the RBM15-mediated m6A modification typically stabilizes target transcripts in other cancers, we provide the first evidence that in BC, this pathway paradoxically triggers ZO2 mRNA decay via YTHDF2 recognition but increases the nuclear accumulation of the remaining ZO2 protein to promote the Snail-mediated EMT. Pharmacological inhibition of METTL3 with STM2457 effectively suppresses this axis in preclinical models, establishing the METTL3/RBM15/ZO2 pathway as a compelling therapeutic target for RBM15-high BC (Fig. 11).

Our findings redefine the functional role of RBM15 in cancer biology, highlighting its context-dependent duality. While previous studies of laryngeal squamous cell carcinoma and hepatocellular carcinoma have consistently shown that the RBM15-mediated m6A modification stabilizes its target transcripts to promote survival [13, 14], our work provides the first evidence of an RBM15-driven, m6A-dependent degradation mechanism. This functional switch establishes RBM15 not only as a generic oncogene but also as a master contextual regulator of m6A topology, whose substrate specificity and functional outcomes are likely governed by tissue-specific cofactors and the local chromatin environment [31, 32]. This context specificity positions RBM15 as a high-value target for precision oncology and warrants further investigation into the molecular determinants underlying its cancer type-specific functions.

Furthermore, we identified a previously unappreciated layer of regulation within the ZO2-Snail-EMT pathway. Our data reveal that while RBM15-mediated degradation reduces total ZO2 protein levels, it paradoxically increases the relative nuclear accumulation of the remaining ZO2 protein. This finding is consistent with the known role of ZO2 as a master regulator of gene expression that can shuttle to the nucleus [21, 33]. Our ChIP-seq and ChIP-qPCR analyses provide conclusive evidence that nuclear ZO2 directly binds to the SNAI1 promoter and acts as a transcriptional activator. These findings answer a key mechanistic question by showing that RBM15 promotes Snail expression and the EMT in a ZO2-dependent manner. This intricate regulation, in which RBM15 controls both the abundance and subcellular localization of a key tumor suppressor [34], adds significant depth to our understanding of EMT regulation in BC. The paradoxical increase in nuclear ZO2 levels despite a global reduction in the ZO2 protein level suggests that the m6A modification may selectively regulate the subcellular localization of ZO2, potentially through effects on nuclear import receptor binding or by protecting ZO2 from degradation pathways in the cytoplasm. Future investigations employing proteomics to identify RBM15-interacting nuclear transport factors and mutagenesis studies of ZO2 itself are warranted to fully dissect this complex regulatory layer.

In our preclinical models, STM2457 exerted potent antitumor effects by broadly suppressing m6A methylation across the transcriptome, resulting in reduced global m6A levels, restored ZO2 expression, and significant inhibition of orthotopic tumor growth and lung metastasis, all of which were associated with a favorable safety profile. These results position pharmacological inhibition of the m6A writer complex as a promising therapeutic strategy for RBM15-high BC, leveraging the inherent promiscuity of global m6A suppression to target multiple oncogenic pathways simultaneously. The therapeutic efficacy of STM2457 is substantially dependent on the integrity of the ZO2 pathway in BC cells, as ZO2 knockdown rendered cancer cells significantly more resistant to the drug, demonstrating that ZO2 restoration is a primary driver of antitumor activity in our model. However, we acknowledge that the widespread suppression of m6A methylation likely engages multiple additional m6A-dependent mechanisms beyond the RBM15/ZO2 axis, contributing to the overall therapeutic benefit. This inherent promiscuity of global m6A inhibition, while potentially advantageous for clinical efficacy because it simultaneously targets multiple pathways, complicates the mechanism and necessitates comprehensive transcriptomic profiling to delineate all therapeutic targets and potential off-target effects.

We investigated the role of the METTL3/RBM15/ZO2 mechanism in two molecularly distinct BC cell lines (T24 and 5637), which represent different molecular subtypes, to assess the generalizability and validate the robustness of our findings. More importantly, these findings were confirmed in clinically relevant patient-derived organoids (PDOs), which better recapitulate the complexity of human tumors [35, 36]. Furthermore, we established a favorable therapeutic window, with STM2457 exhibiting more than 10-fold greater selectivity for cancer cells than for nonmalignant urothelial cells, supporting the clinical translational potential of this approach.

Beyond direct cytotoxicity, an exciting opportunity lies in combining m6A inhibition with immunotherapy. The EMT is increasingly recognized as a driver of immune evasion, with mesenchymal cells exhibiting reduced antigen presentation and increased expression of immunosuppressive molecules [37, 38]. Emerging evidence indicates that METTL3 acts as a key architect of the immunosuppressive tumor microenvironment [39] partly by stabilizing the PD-L1 mRNA via the m6A modification, thereby facilitating immune evasion [40, 41]. Therefore, STM2457 may have dual therapeutic benefits: (1) direct anti-tumor activity via ZO2 restoration and EMT suppression and (2) immunomodulatory effects by reducing PD-L1 expression and potentially reversing immune evasion. Given that the RBM15/ZO2 axis drives EMT—a process intrinsically linked to immune exclusion and resistance to immune checkpoint blockade—targeting this pathway could enhance the sensitivity of BC to immunotherapies by reversing EMT. Future studies should therefore focus on evaluating the synergy between STM2457 and anti-PD-1/PD-L1 antibodies in immunocompetent preclinical models and assessing changes in immune cell infiltration, T-cell activation, and the composition of the tumor microenvironment. These investigations are critical for advancing m6A-targeted therapies toward clinical application and establishing a rational strategy to overcome immunotherapy and chemotherapy resistance in BC.

Several limitations in this study warrant acknowledgment. First, stratified analyses based on molecular subtypes (e.g., FGFR3 or TP53 mutations) are highly valuable. Given that the role of RBM15 may vary across distinct molecular contexts, future prospective studies should integrate RBM15 expression with comprehensive genomic profiling. This approach will help assess its independent prognostic value beyond that of established classification systems and determine whether the METTL3/RBM15/ZO2 axis is consistently active across all BC subtypes.

Second, our reliance on immunodeficient mouse models limits the assessment of the RBM15/m6A axis in the context of host immunity. Given our proposal to combine m6A inhibition with immunotherapy, syngeneic and humanized mouse models are essential to validate the immunomodulatory effects of targeting this pathway and to assess the efficacy of combination therapies with immune checkpoint inhibitors. These models will be critical for understanding the interplay between tumor-intrinsic m6A signaling and the immune microenvironment.

Third, while we demonstrated that RBM15-mediated ZO2 degradation paradoxically increases its nuclear accumulation, the precise molecular mechanism underlying this subcellular redistribution remains elusive. Whether RBM15 directly facilitates nuclear import through interactions with transport receptors, whether m6A modification affects compartmentalized translation, and whether selective protection from cytoplasmic degradation is involved require further investigation through proteomics, structural studies, and live-cell imaging approaches.

An additional limitation of this study is the use of a single-site m6A point mutation targeting the GGACU motif at chr9:69221025. Although this site harbors the strongest m6A enrichment signal and represents the canonical DRACH consensus sequence, the ZO2 3’UTR contains multiple potential m6A modification sites. While our single-site mutation effectively established the critical role of this primary m6A site, a comprehensive understanding of the m6A regulatory landscape in the ZO2 mRNA will require future investigations of combinatorial mutations affecting multiple m6A motifs.

Finally, STM2457 exerts broad antitumor effects through widespread m6A suppression across the transcriptome, not exclusively via the RBM15/ZO2 axis. While our ZO2 rescue experiments demonstrated the primary contribution of this pathway, the therapeutic benefits likely involve multiple m6A-dependent mechanisms. Future genome-wide approaches (m6A-seq, RNA-seq, and ribosome profiling) and unbiased proteomic profiling of RBM15-interacting proteins will be essential for mapping complete therapeutic targets, identifying biomarkers predicting patient responses, and discovering additional regulatory cofactors.

## Conclusions

In conclusion, this study establishes the METTL3/RBM15/ZO2 axis as a novel and therapeutically tractable driver of BC progression. The context-specific degradation mechanism mediated by RBM15, coupled with its paradoxical enhancement of nuclear ZO2 localization, represents a previously unrecognized layer of m6A-dependent regulation in cancer. Our findings support the clinical development of METTL3 inhibitors for RBM15-high BC, particularly in combination with immunotherapy, and highlight the importance of understanding m6A biology in a tissue- and cancer-type-specific context for precision oncology. 

## Supplementary Information

Supplementary Material 1. Additional file 1.

Supplementary Material 2. Additional file 2.

## Data Availability

The datasets used and/or analyzed during the current study are available from the corresponding author upon reasonable request.

## Abbreviations

3'UTR: 3’ Untranslated Region
ANOVA: Analysis of Variance
ATCC: American Type Culture Collection
BC: Bladder cancer
BSA: Bovine serum albumin
CCK-8: Cell Counting Kit-8
cDNA: complementary deoxyribonucleic acid
CDS: Coding DNA sequence
ChIP: Chromatin immunoprecipitation
ChIRP: Chromatin isolation by RNA purification
Co-IP: Coimmunoprecipitation
DCA: Decision curve analysis
DEGs: Differentially expressed genes
DFS: Disease-free survival
EMT: Epithelial–mesenchymal transition
FDR: False discovery rate
GEO: Gene Expression Omnibus
GEPIA2: Gene Expression Profiling Interactive Analysis 2
GSEA: Gene set enrichment analysis
GTEx: Genotype–Tissue Expression
HCC: Hepatocellular carcinoma
IC50: Half-maximal inhibitory concentration
IF: Immunofluorescence
IGV: Integrative Genomics Viewer
IHC: Immunohistochemistry
IRS: Immunoreactivity score
KO: Knockout
LSCC: Laryngeal squamous cell carcinoma
m6A: N6-methyladenosine
m6A-IP–qPCR: m6A RNA Immunoprecipitation–qPCR
MeRIP-seq: m6A RNA Immunoprecipitation Sequencing
METTL14: Methyltransferase 14
METTL3: Methyltransferase 3
MIBC: Muscle-Invasive Bladder Cancer
MOI: Multiplicity of Infection
mRNA: messenger Ribonucleic Acid
MUT: Mutant
NMIBC: Non-Muscle-Invasive Bladder Cancer
OS: Overall survival
PDOs: Patient-derived organoids
PFA: Paraformaldehyde
qPCR: quantitative real-time Polymerase Chain Reaction
RBM15: RNA Binding Motif Protein 15
RIP–qPCR: RNA Immunoprecipitation–qPCR
RIP-seq: RNA Immunoprecipitation Sequencing
RNA-seq: RNA sequencing
RT: Room temperature
SD: Standard deviation
sgRNAs: single guide RNAs
shRNA: short hairpin RNA
STR: Short Tandem Repeat
T7E1: T7 Endonuclease 1
TCGA: The Cancer Genome Atlas
WB: Western blot
WT: Wild-Type
WTAP: Wilms’ Tumor 1-Associating Protein
YTHDF2: YTH N(6)-Methyladenosine RNA Binding Protein 2
ZO2: Zona Occludens 2
