# Supplementary material for: RBM15 drives bladder cancer progression through YTHDF2-dependent m6A-mediated regulation of ZO2
Source: J Exp Clin Cancer Res. 2026 Mar 30;45:118. doi: 10.1186/s13046-026-03684-9 (PMC13174013; doi:10.1186/s13046-026-03684-9)
Supplement: Supplementary file 1 — Supplementary Material 1. Additional file 1. [file 13046_2026_3684_MOESM1_ESM.pdf]

RBM15 drives bladder cancer progression through YTHDF2-dependent m6A-mediated regulation of ZO2

Yuhui He[1,2,3,4,#], Yanqing Gong[1,2,3,#], Yucai Wu[1,2,3,#], Shiming He[1,2,3], Yang Wang[5,6], Wenzhi Gao[1,2,3], Tai Tian[1,2,3], Xinyu Xu[5,6], Liqun Zhou[1,2,3], Zhenduo Shi[7,8], Conghui Han[7,8], Ninghan Feng[5,6,*], Jianfeng Wang[4,*], Xuesong Li[1,2,3,*]

[1]Department of Urology, Peking University First Hospital, Beijing 100034, China
[2]Institute of Urology, Peking University, Beijing 100034, China
[3]Beijing Key Laboratory of Urogenital Diseases (Male) Molecular Diagnosis and Treatment Center, Beijing 100034, China
[4]Department of Urology, China-Japan Friendship Hospital, Beijing 100029, China
[5]Department of Urology, Jiangnan University Medical Center, Wuxi 214122, China
[6]Department of Urology, Wuxi No. 2 People's Hospital, Medical School of Nantong University, Wuxi 214002, China
[7]Department of Urology, Xuzhou Central Hospital, Southeast University, Xuzhou 221000, China
[8]Department of Urology, Xuzhou Clinical School of Xuzhou Medical University, Xuzhou 221004, China

[#] Yuhui He, Yanqing Gong and Yucai Wu contributed equally to this work.

***Corresponding authors**

Xuesong Li, Department of Urology, Peking University First Hospital, Beijing 100034, China. E-mail: pineneedle@sina.com; Jianfeng Wang, Department of Urology, China-Japan Friendship Hospital, Beijing 100029, China. E-mail: zryhyy1@126.com; Ninghan Feng, Department of Urology, Jiangnan University Medical Center, Wuxi 214122, China. E-mail: n.feng@jiangnan.edu.cn

Additional file 1. Supplementary Figures

Fig. S1

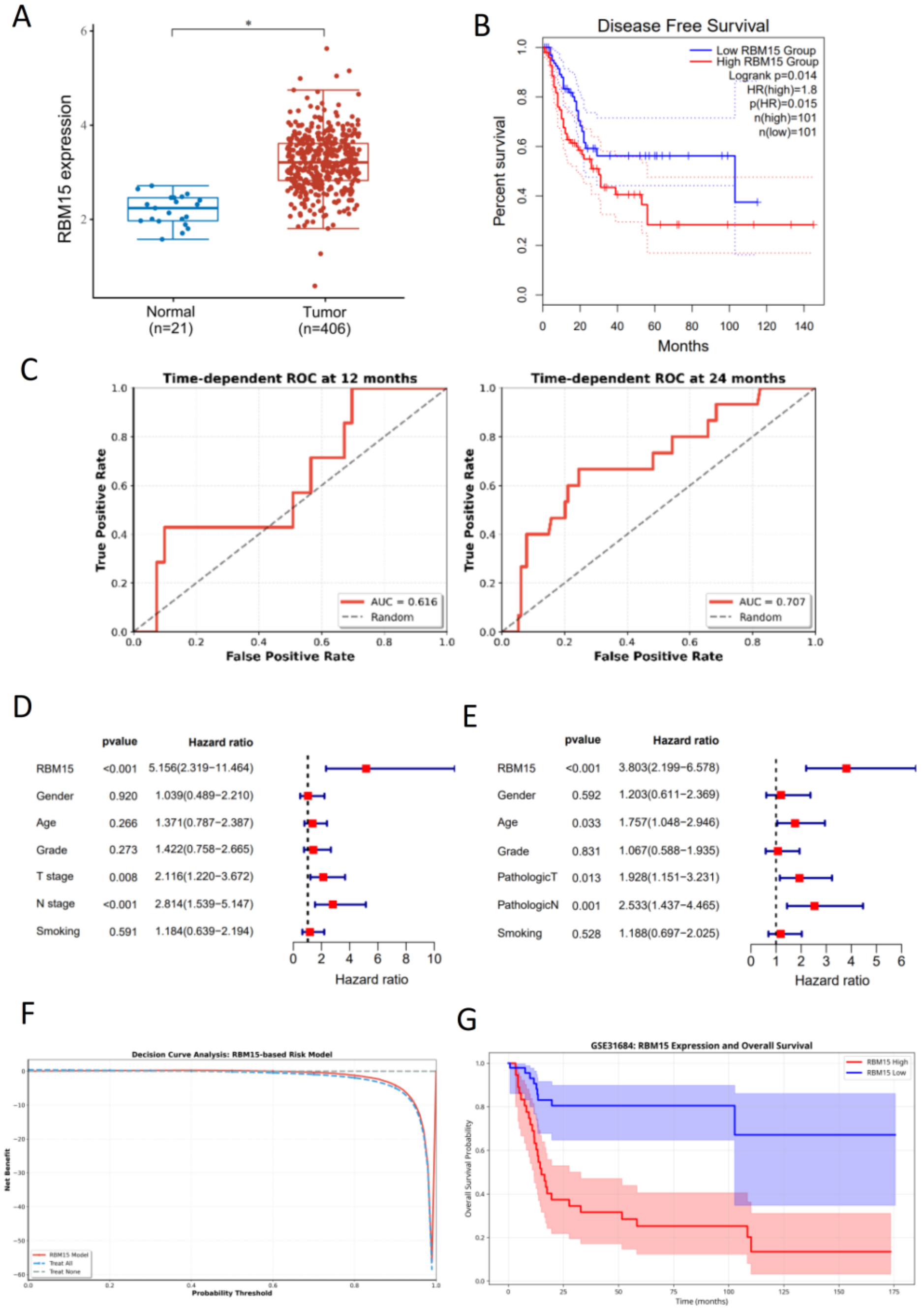

Fig. S1. RBM15 is upregulated in BC and correlates with poor prognosis (Supplementary). A. RBM15 mRNA expression in BC (n = 408) versus normal tissues (n = 21) from the combined TCGA-BLCA and GTEx datasets. **B.** Kaplan-Meier analysis of Disease-Free Survival (DFS) based on RBM15 expression in the TCGA-BLCA cohort. (n = 408). **C.** Time-dependent ROC curve analysis for the RBM15-based prognostic model at 12 and 24 months, showing AUC values of 0.616 and 0.707, respectively. **D-E.** Forest plots of multivariate Cox regression analysis for (C) 6-year and (D) 13-year overall survival in the PKU cohort. **F.** Decision Curve Analysis (DCA) for the RBM15-

based risk model, demonstrating the net clinical benefit of using the model for treatment decisions compared to the "treat all" or "treat none" strategies across a range of probability thresholds. **G.** Kaplan-Meier analysis of Overall Survival (OS) based on RBM15 expression in the GSE31684 cohort (n = 88).

Fig. S2

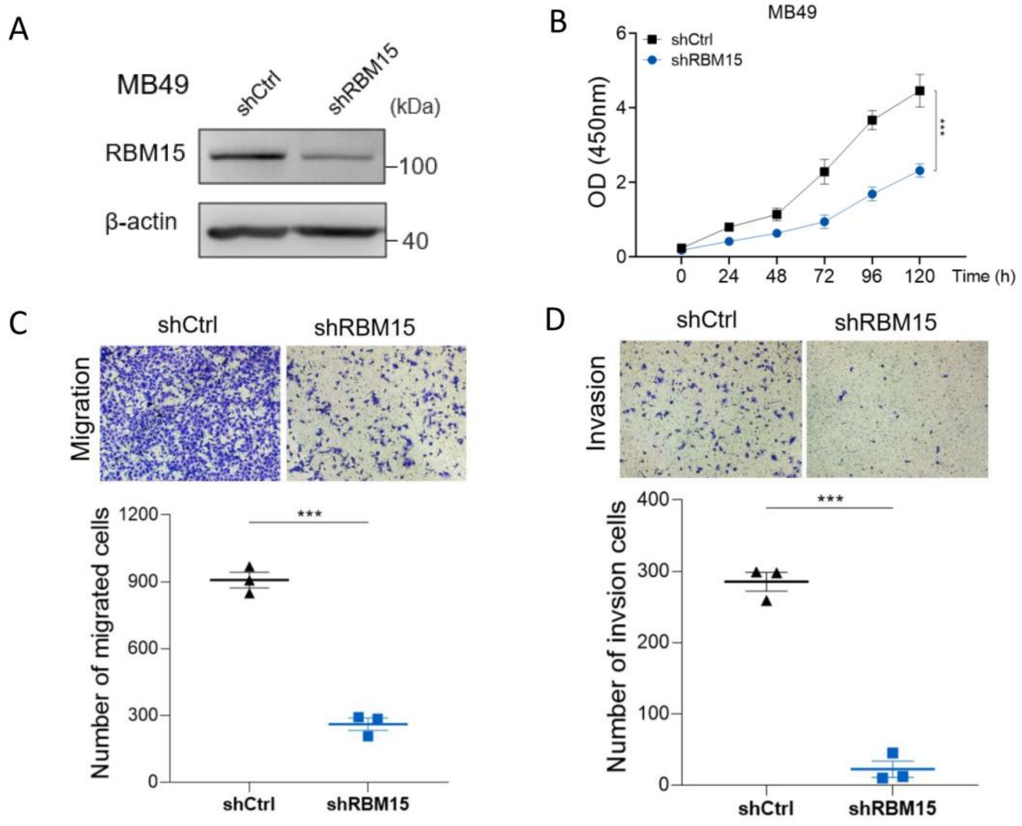

Fig. S2. RBM15 knockdown impairs malignant phenotypes in murine BC cells. A. WB confirming efficient RBM15 knockdown in MB49 cells. **B.** CCK-8 assay demonstrating that RBM15 knockdown significantly inhibited MB49 cell proliferation. **C, D.** Transwell assays showing that RBM15 knockdown markedly suppressed the migration (C) and invasion (D) capacities of MB49 cells. Scale bars: 100× (10× objective × 10× ocular). Three randomly selected fields per sample (100× magnification) were quantified. ***$p < 0.001$.

Fig. S3

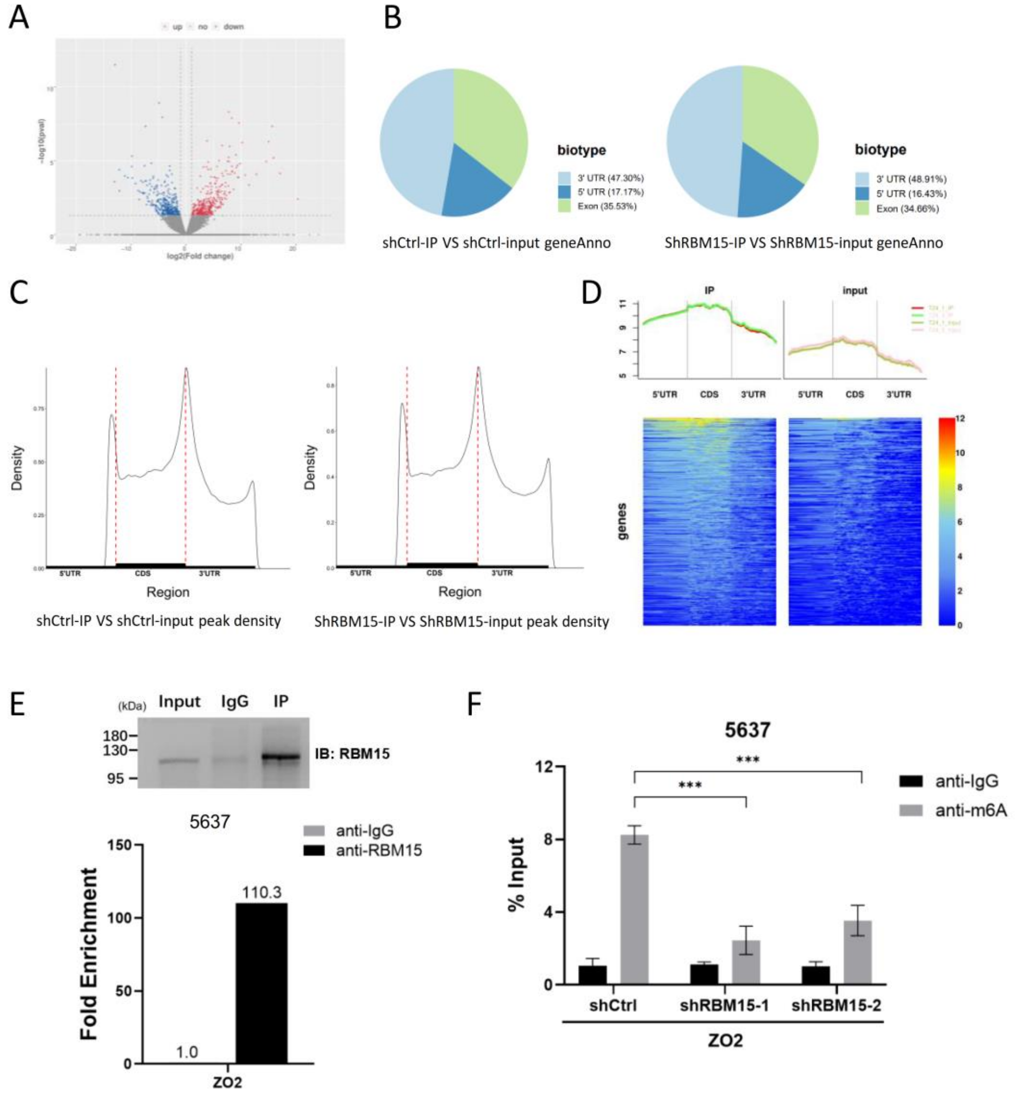

Fig. S3 Multi-omics Analysis to Identify RBM15 Targets. A. Volcano plot of differentially expressed genes from RNA-seq analysis. **B.** Pie charts showing the genomic distribution of m6A peaks in control and RBM15-knockdown cells. **C, D.** Metagene profiles (C) and heatmaps (D) showing the distribution of RBM15 binding peaks across gene bodies. **E.** RIP-qPCR analysis confirming the direct interaction between RBM15 and ZO2 mRNA in 5637 cells. **F.** m6A-IP-qPCR analysis showing reduced m6A levels on ZO2 mRNA upon RBM15 knockdown in 5637 cells. ***$p < 0.001$.

Fig. S4

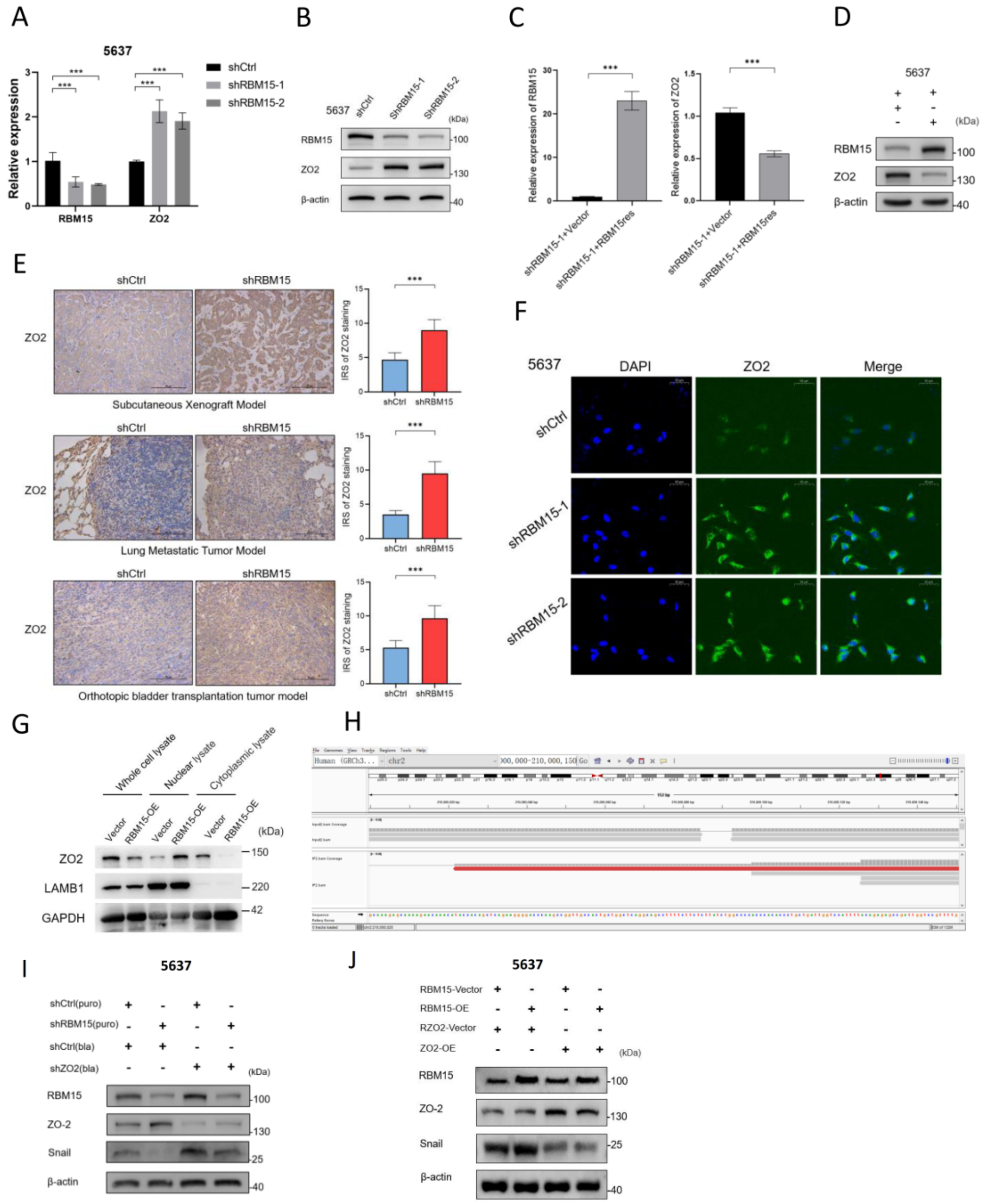

Fig. S4. RBM15 negatively regulates ZO2 expression and drives nuclear localization of ZO2. A, B. qPCR (A) and WB (B) analysis of RBM15 and ZO2 expression in 5637 cells following RBM15 knockdown. **C, D.** qPCR (C) and WB (D) analysis of ZO2 expression in RBM15-knockdown 5637 cells after re-expression of RBM15. **E.** IHC staining and quantitative analysis of ZO2 in tumor tissues from shCtrl and shRBM15 groups in subcutaneous xenograft, lung metastasis, and orthotopic BC transplantation models. Scale bar: 20 μm. **F.** IF staining of ZO2 (green) in 5637 cells after RBM15 knockdown. Nuclei were counterstained with DAPI (blue). Scale bar: 10 μm. **G.** WB analysis of ZO2 protein levels in nuclear and cytoplasmic fractions from 5637 cells overexpressing RBM15. **H.** IGV snapshot of ChIP-seq data showing the binding peak of ZO2 at the promoter region of the SNAI1P1 pseudogene. **I.** WB analysis of RBM15, ZO2, and Snail protein expression in 5637 cells with single or double knockdown of RBM15 and/or ZO2. **J.** WB analysis

of RBM15, ZO2, and Snail protein expression in 5637 cells with single or co-overexpression of RBM15 and/or ZO2. ***$p < 0.001$.

Fig. S5

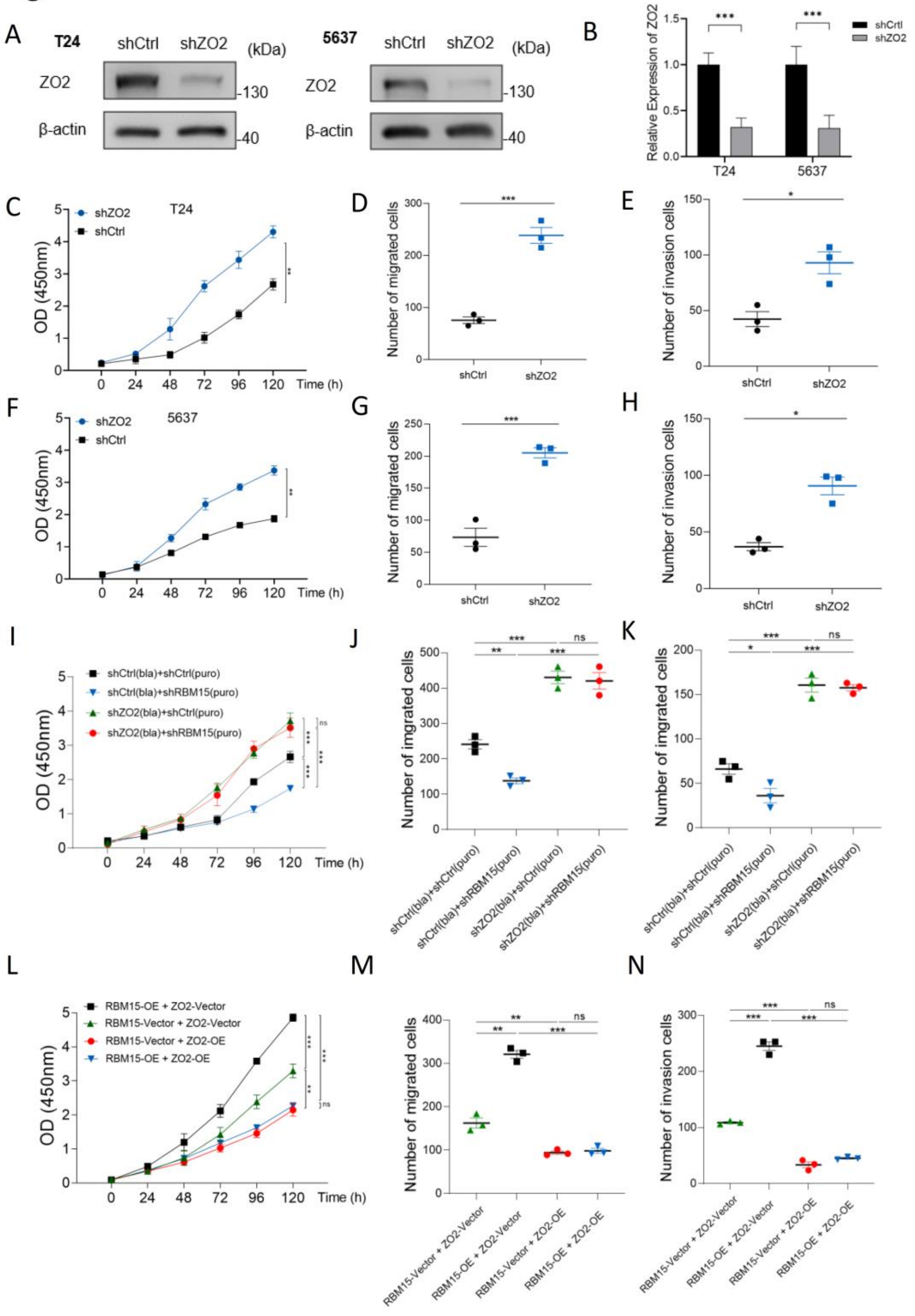

Fig. S5. ZO2 functions as a tumor suppressor and mediates the oncogenic effects of RBM15 in BC cells. **A, B.** Western blot (A) and qPCR (B) analysis confirming the efficient knockdown of ZO2 in T24 and 5637 cells. **C-H.** Knockdown of ZO2 promoted cell proliferation (C, F), migration

(D, G), and invasion (E, H) in both T24 and 5637 cells. **I-K.** In 5637 cells, the anti-tumor effects of RBM15 knockdown on proliferation (I), migration (J), and invasion (K) were reversed by the concurrent knockdown of ZO2. **L-N.** In 5637 cells, the pro-tumorigenic effects of RBM15 overexpression on proliferation (L), migration (M), and invasion (N) were suppressed by the co-overexpression of ZO2. *$p < 0.05$, **$p < 0.01$, ***$p < 0.001$, ns, $p > 0.05$

Fig. S6

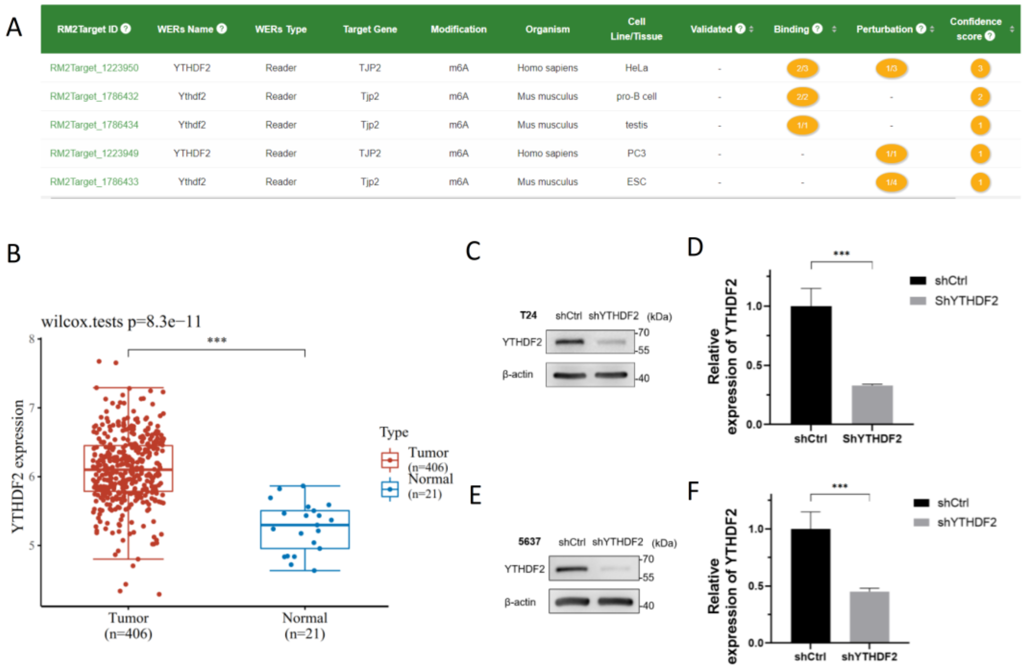

Fig. S6. YTHDF2 is a potential m6A reader for ZO2 and is upregulated in BC. A. Prediction of YTHDF2 as an m6A reader for ZO2 (TJP2) from the m6A2Target database. **B.** Analysis of TCGA and GTEx datasets shows significant upregulation of YTHDF2 expression in bladder tumor tissues ($n = 406$) compared to normal tissues ($n = 21$). **C-F.** WB (C, E) and qPCR (D, F) analysis confirming the efficient knockdown of YTHDF2 in T24 and 5637 cells, respectively. ***$p < 0.001$

Fig. S7

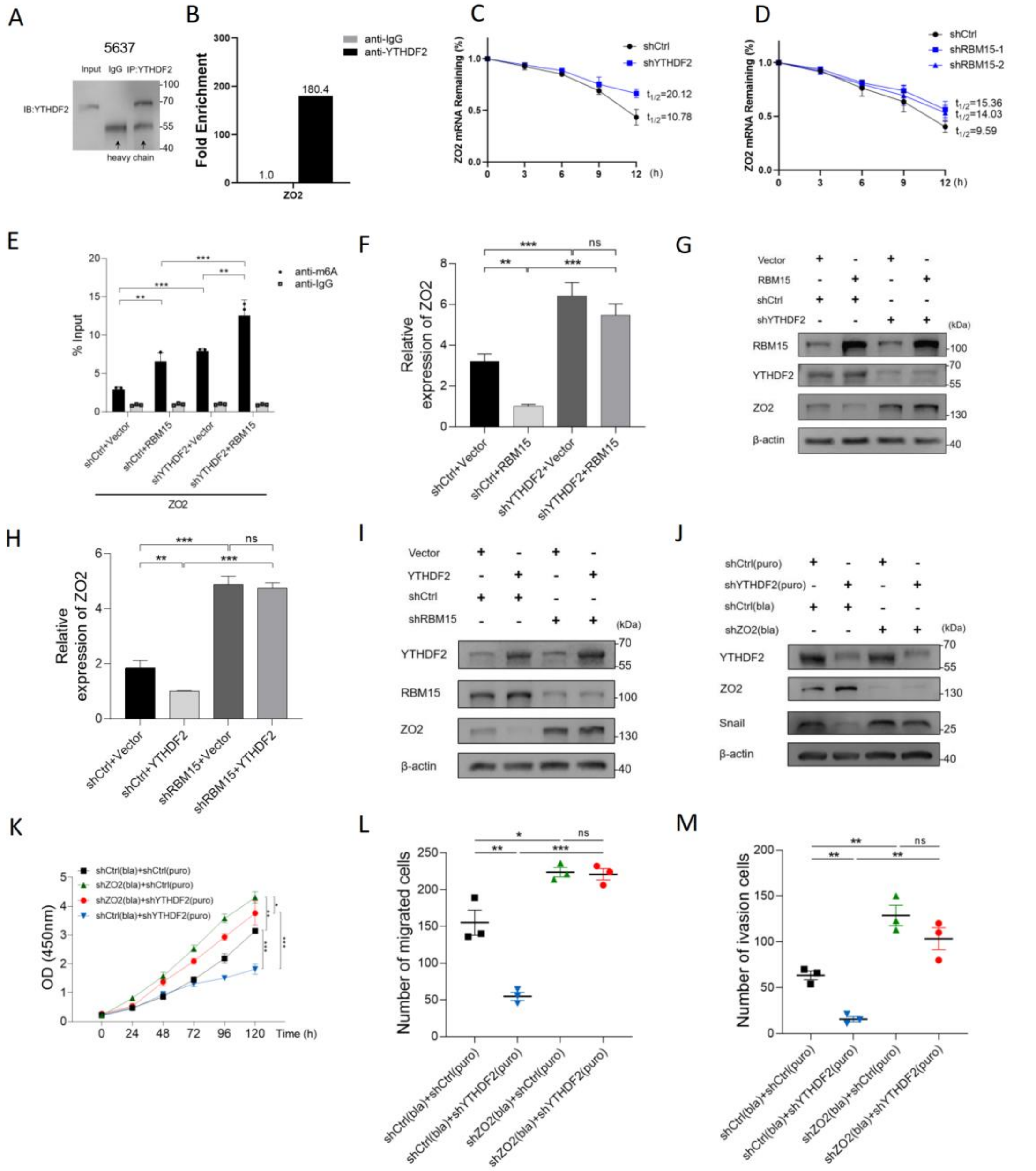

Fig. S7. Validation of the RBM15-YTHDF2-ZO2 axis in 5637 cells. A, B. RIP-qPCR confirms the direct binding of YTHDF2 to ZO2 mRNA in 5637 cells. **C, D.** mRNA decay assays demonstrate that knockdown of YTHDF2 (C) or RBM15 (D) prolongs the half-life of ZO2 mRNA. **E.** m6A-RIP-qPCR shows that RBM15 overexpression enhances ZO2 m6A levels, which is reversed by YTHDF2 knockdown. **F, G.** The downregulation of ZO2 mRNA (F) and protein (G) by RBM15 overexpression is rescued by YTHDF2 knockdown. **H, I.** The suppression of ZO2 mRNA (H) and protein (I) by YTHDF2 overexpression is abolished by RBM15 knockdown. **J-M**. The anti-tumor effects of YTHDF2 knockdown on EMT marker Snail expression (J), proliferation (K), migration (L), and invasion (M) are all rescued by the simultaneous knockdown of ZO2. *$p < 0.05$, **$p < 0.01$, ***$p < 0.001$, ns, $p > 0.05$

Fig. S8

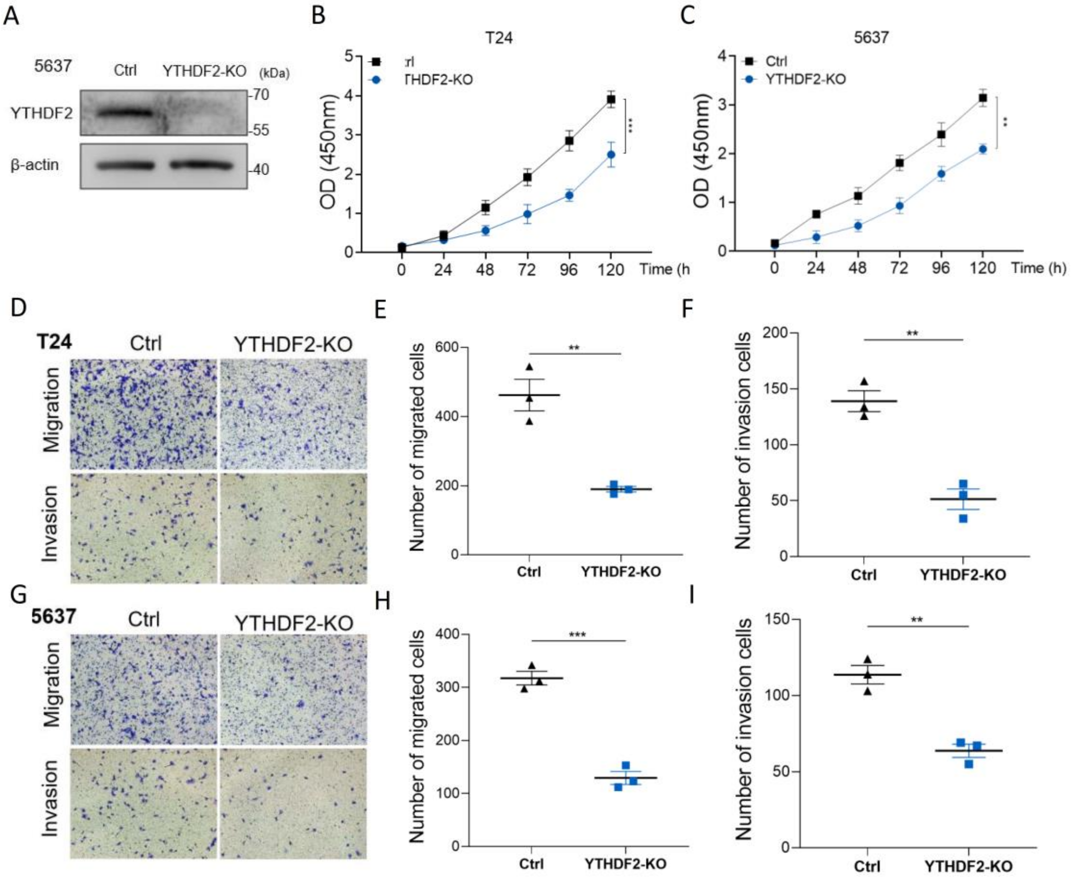

Fig. S8. YTHDF2 knockout suppresses malignant phenotypes in BC cells. A. WB confirming YTHDF2 knockout in 5637 cells. **B, C.** CCK-8 assays showing YTHDF2 knockout suppressed proliferation in T24 (B) and 5637 (C) cells. **D.** Representative Transwell migration/invasion images in T24 cells. **E, F.** Quantification showing reduced migration (E) and invasion (F) in YTHDF2-KO T24 cells. **G.** Representative Transwell images in 5637 cells. **H, I.** Quantification confirming reduced migration (H) and invasion (I) in YTHDF2-KO 5637 cells. $^{**}p < 0.01$, $^{***}p < 0.001$

Fig. S9

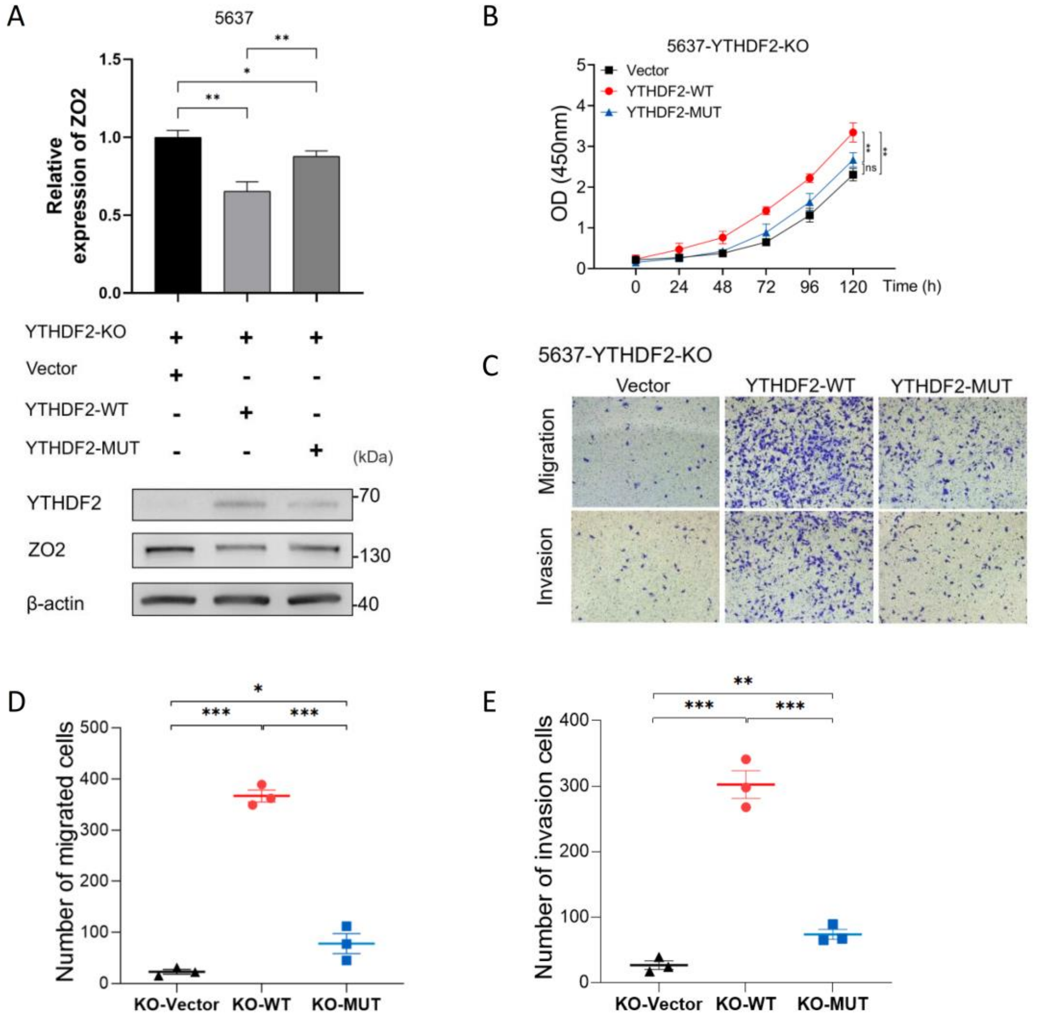

Fig. S9. The m6A-binding activity of YTHDF2 is essential for its oncogenic function in 5637 cells. A. ZO2 expression (qPCR, upper; Western blot, lower) in YTHDF2-KO 5637 cells reconstituted with indicated constructs. YTHDF2-WT suppressed ZO2; YTHDF2-MUT showed marginal effect. **B.** CCK-8 assay. YTHDF2-WT rescued proliferation; YTHDF2-MUT showed no significant rescue. **C.** Representative Transwell migration/invasion images. **D, E.** Quantification of migrated (D) and invaded (E) cells. YTHDF2-WT restored migration/invasion; YTHDF2-MUT showed ~20% of WT effect. *$p < 0.05$, **$p < 0.01$, ***$p < 0.001$

Fig. S10

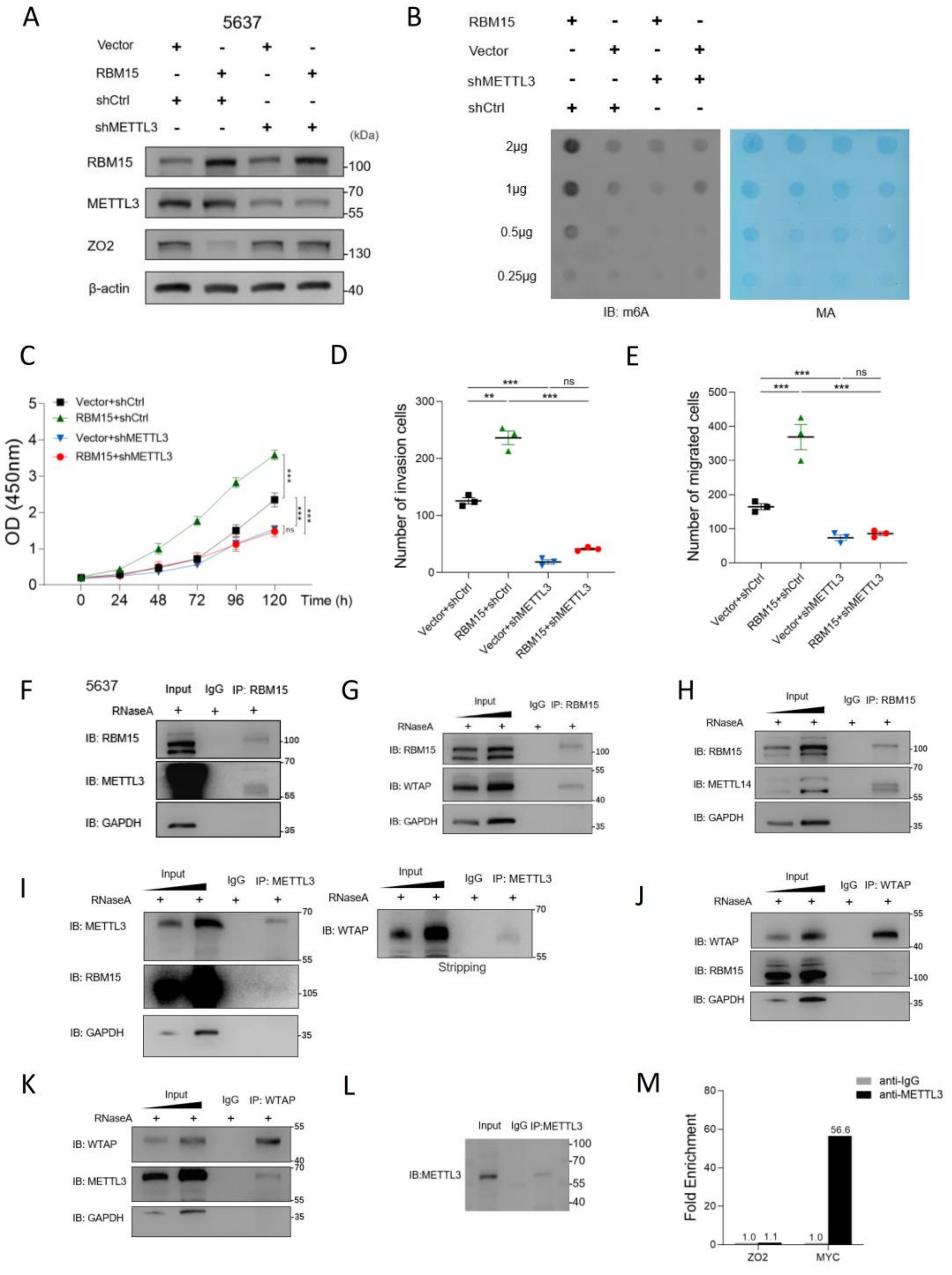

Fig. S10. RBM15 drives m6A hypermethylation and oncogenesis via collaboration with the METTL3-WTAP-METTL14 methyltransferase complex (Supplementary in 5637 cells). A, B. METTL3 is essential for RBM15-mediated ZO2 suppression and m6A deposition in 5637 cells. **A.** METTL3 knockdown abolished RBM15 overexpression-induced ZO2 downregulation. **B.** Global m6A reduction by METTL3 knockdown was not rescued by RBM15 overexpression. MA, methylene blue. **C-E.** RBM15 functional dependency on METTL3 in 5637 cells. RBM15 OE failed to reverse METTL3 knockdown-induced suppression of proliferation (**C**), migration (**D**), and invasion (**E**). **F-K.** Core methyltransferase complex interactions between RBM15 and METTL3-

WTAP-METTL14 in 5637 cells. co-immunoprecipitation lysates revealed RBM15 binding to METTL3 (**F**), METTL14 (**G**), and WTAP (**H**). Reciprocal interactions: METTL3-WTAP (**I** left panel), METTL3-RBM15 (**I** right panel); WTAP-RBM15 (**J**), WTAP-METTL3 (**K**). **L-M.** METTL3 does not directly bind ZO2 mRNA in 5637 cells. RIP-qPCR showed no enrichment of ZO2 mRNA in METTL3 pulldowns, contrasting with MYC (positive control). $^{**}p < 0.01$, $^{***}p < 0.001$, ns, $p > 0.05$

Fig. S11

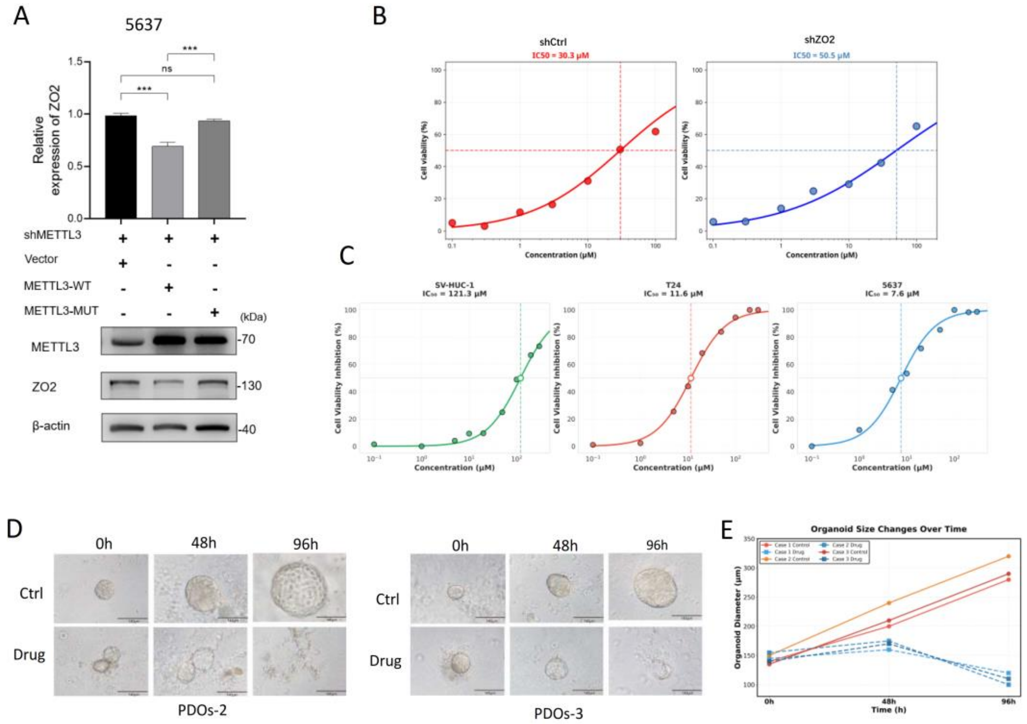

Fig. S11. METTL3 catalytic activity is required for ZO2 regulation, and ZO2 mediates STM2457 sensitivity in BC cells. A. qPCR (upper) and WB (lower) of ZO2 in METTL3-knockdown 5637 cells reconstituted with Vector, METTL3-WT, or METTL3-D395A. Only METTL3-WT suppresses ZO2 expression; METTL3-D395A fails to restore ZO2 suppression. β-actin, loading control. **B.** Cell viability curves and IC50 values for STM2457 in shCtrl and shZO2 T24 cells. ZO2 knockdown increases IC50 from 10.3 μM to 30.5 μM (~3-fold), indicating ZO2-dependent drug sensitivity. **C.** Cell viability curves comparing STM2457 sensitivity in non-malignant SV-HUC-1 cells versus T24 and 5637 cancer cells. IC50 values: SV-HUC-1, 121.3 μM; T24, 11.6 μM; 5637, 7.6 μM, demonstrating >10-fold selectivity for cancer cells. **D.** Representative images of PDO-2 and PDO-3 treated with STM2457 (10 μM) at 0, 48, and 96 h. STM2457 inhibits organoid growth in both models. **E.** Quantification of organoid size changes over time in three independent PDOs. STM2457 treatment significantly reduces organoid growth compared to controls. $^{***}p < 0.001$.

Fig. S12

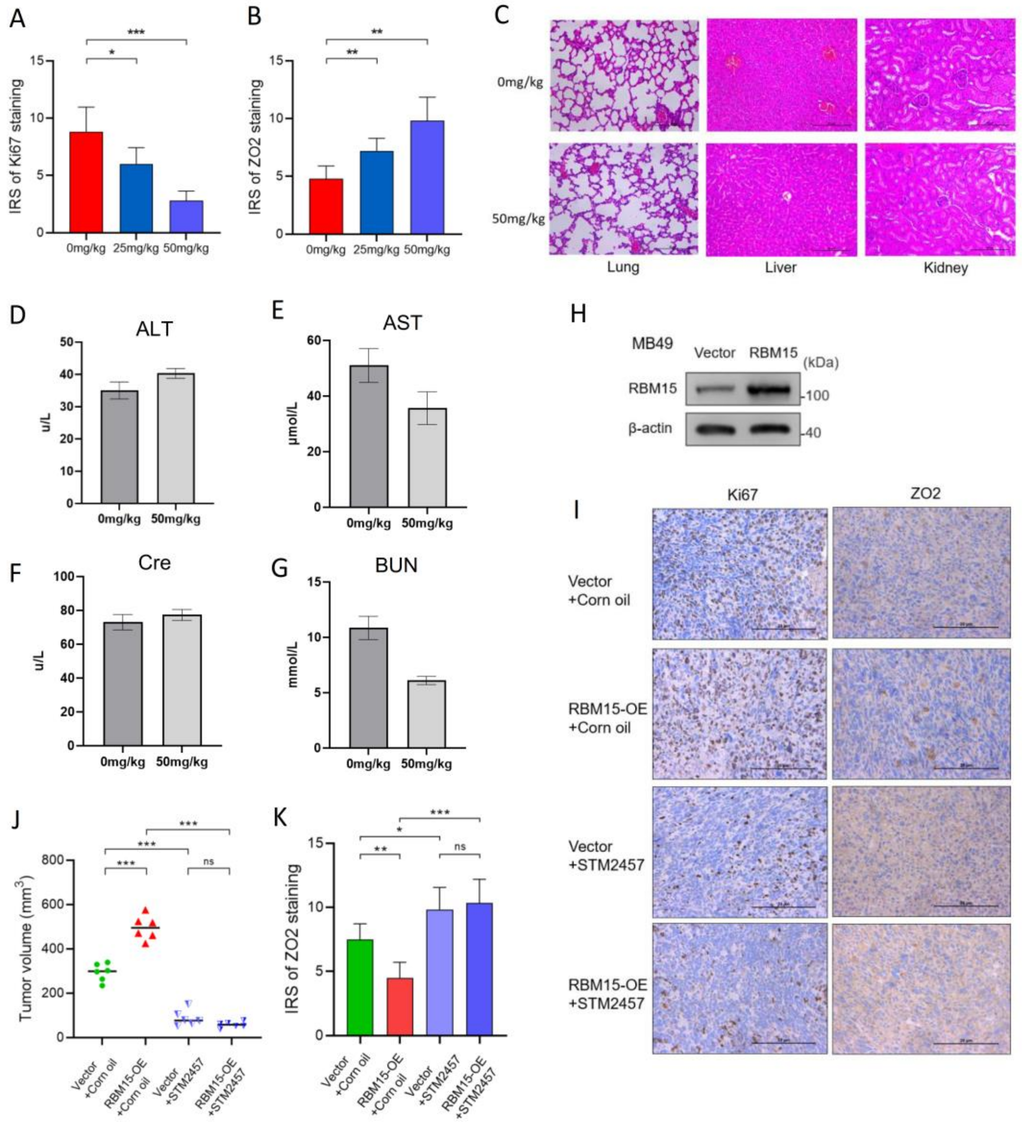

Fig. S12. STM2457 restores ZO2 expression without systemic toxicity and reverses RBM15-driven tumor progression. A. Ki-67 IRS quantification in orthotopic tumors showing dose-dependent reduction in proliferation with STM2457 treatment. **B.** ZO2 IRS quantification showing dose-dependent restoration of ZO2 expression in tumors. **C.** Representative HE staining of lung, liver, and kidney from mice treated with vehicle (0 mg/kg) or STM2457 (50 mg/kg). No histological abnormalities observed. **D–G.** Serum biochemistry analysis: ALT (D), AST (E), creatinine (F), and BUN (G) levels showing no significant differences between vehicle and STM2457-treated groups, confirming absence of hepatic or renal toxicity. **H.** WB confirming RBM15 overexpression in MB49 cells. **I.** Representative IHC images of Ki67 and ZO2 staining in orthotopic tumors from Vector/RBM15-OE mice treated with corn oil or STM2457. STM2457 reduces Ki67 and restores ZO2 expression in both groups. **J.** Tumor volume quantification in the RBM15-OE orthotopic model. STM2457 significantly reduces tumor volume regardless of RBM15 status. **K.** ZO2 IRS quantification confirming STM2457-mediated ZO2 restoration in RBM15-OE tumors. *$p < 0.05$, **$p < 0.01$, ***$p < 0.001$, ns, $p > 0.05$
