# Supplementary material for: RBM15 drives bladder cancer progression through YTHDF2-dependent m6A-mediated regulation of ZO2
Source: J Exp Clin Cancer Res. 2026 Mar 30;45:118. doi: 10.1186/s13046-026-03684-9 (PMC13174013; doi:10.1186/s13046-026-03684-9)
Supplement: Supplementary file 2 — Supplementary Material 2. Additional file 2. [file 13046_2026_3684_MOESM2_ESM.pdf]

RBM15 drives bladder cancer progression through YTHDF2-dependent m6A-mediated regulation of ZO2

Yuhui He[1,2,3,4,#], Yanqing Gong[1,2,3,#], Yucai Wu[1,2,3,#], Shiming He[1,2,3], Yang Wang[5,6], Wenzhi Gao[1,2,3], Tai Tian[1,2,3], Xinyu Xu[5,6], Liqun Zhou[1,2,3], Zhenduo Shi[7,8], Conghui Han[7,8], Ninghan Feng[5,6,*], Jianfeng Wang[4,*], Xuesong Li[1,2,3,*]

[1]Department of Urology, Peking University First Hospital, Beijing 100034, China
[2]Institute of Urology, Peking University, Beijing 100034, China
[3]Beijing Key Laboratory of Urogenital Diseases (Male) Molecular Diagnosis and Treatment Center, Beijing 100034, China
[4]Department of Urology, China-Japan Friendship Hospital, Beijing 100029, China
[5]Department of Urology, Jiangnan University Medical Center, Wuxi 214122, China
[6]Department of Urology, Wuxi No. 2 People's Hospital, Medical School of Nantong University, Wuxi 214002, China
[7]Department of Urology, Xuzhou Central Hospital, Southeast University, Xuzhou 221000, China
[8]Department of Urology, Xuzhou Clinical School of Xuzhou Medical University, Xuzhou 221004, China

[#] Yuhui He, Yanqing Gong and Yucai Wu contributed equally to this work.

***Corresponding authors**

Xuesong Li, Department of Urology, Peking University First Hospital, Beijing 100034, China. E-mail: pineneedle@sina.com; Jianfeng Wang, Department of Urology, China-Japan Friendship Hospital, Beijing 100029, China. E-mail: zryhyy1@126.com; Ninghan Feng, Department of Urology, Jiangnan University Medical Center, Wuxi 214122, China. E-mail: n.feng@jiangnan.edu.cn

Additional file 2. Supplementary Tables

Table S1: Summary of m6A-related functions of RBM15 in different cancers.

Cancer Type	Target Gene/RNA	m6A Reader	Effect of RBM15-mediated m6A on Target RNA	Cellular/Phenotypic Effect	Reference(s)
Laryngeal Squamous Cell Carcinoma (LSCC)	TMBIM6	IGF2BP3	Stabilization	Promotes migration and invasion	[1]
Hepatocellular Carcinoma (HCC)	YES1	IGF2BP1	Stabilization	Promotes malignant progression	[2]
Clear Cell Renal Cell Carcinoma (ccRCC)	CXCL11	Not Identified	Stabilization	Promotes proliferation, migration, invasion, and macrophage infiltration	[3]
Colorectal Cancer (CRC)	MyD88	Not Identified	Stabilization (Inferred)	Promotes proliferation and invasion	[4]
Ovarian Cancer (OC)	UBA6 (via LncRNA UBA6-AS1)	IGF2BP1	Stabilization	Inhibits malignant progression	[5]
Bladder Cancer (BC)	ZO2	YTHDF2	Degradation	Promotes proliferation, migration, and invasion	This study

References:

[1] Wang X, Tian L, Li Y, Wang J, Yan B, Yang L, Li Q, Zhao R, Liu M, Wang P, Sun Y. RBM15 facilitates laryngeal squamous cell carcinoma progression by regulating TMBIM6 stability through IGF2BP3 dependent. J Exp Clin Cancer Res, 2021,40(1):80.

[2] Cai X, Chen Y, Man D, Yang B, Feng X, Zhang D, Chen J, Wu J. RBM15 promotes hepatocellular carcinoma progression by regulating N6-methyladenosine modification of YES1 mRNA in an IGF2BP1-dependent manner. Cell Death Discov, 2021,7(1):315.

[3] Zeng X, Chen K, Li L, Tian J, Ruan W, Hu Z, Peng D, Chen Z. Epigenetic activation of RBM15 promotes clear cell renal cell carcinoma growth, metastasis and macrophage infiltration by regulating the m6A modification of CXCL11. Free Radic Biol Med, 2022,184:135-147.

[4] Zhang Z, Mei Y, Hou M. Knockdown RBM15 Inhibits Colorectal Cancer Cell Proliferation

and Metastasis Via N6-Methyladenosine (m6A) Modification of MyD88 mRNA. Cancer Biother Radiopharm, 2022,37(10):976-986.
[5] Wang Y, Chen Z. Long noncoding RNA UBA6-AS1 inhibits the malignancy of ovarian cancer cells via suppressing the decay of UBA6 mRNA. Bioengineered, 2022,13(1):178-189.

Table S2. Demographic and clinical parameters in Peking University First Hospital bladder cancer cohort.

Characteristics	Overall
Total	137
Sex, n (%)	
Male	114 (83.2%)
Female	23 (16.8%)
Age, mean ± sd	64.832 ± 11.154
BMI, mean ± sd	24.209 ± 3.4035
T stage, n (%)	
T1	39 (28.5%)
T2	40 (29.2%)
T3	26 (19%)
T4	32 (23.4%)
G grade, n (%)	
G2	35 (25.5%)
G3	102 (74.5%)
Smoke, n (%)	
No	95 (69.3%)
Yes	42 (30.7%)
6-year follow-up (129/137 retained)	
Survival	77
Death	52
13-year follow-up (94/137 retained)	
Survival	32
Death	62

Table S3. Experimental cells and culture conditions.

Cell line	Culture conditions
SV-HUC-1	Ham's F-12K + 10% FBS + 1% P/S
T24	1640 + 10% FBS + 1% P/S
5637	1640 + 10% FBS + 1% P/S
MB49	DMEM + 10% FBS + 1% P/S
HEK-293T	DMEM + 10% FBS + 1% P/S

Table S4. Experimental correlation primers and their sequences.

Gene	Application	Sequence (5'→3')	
RBM15	qPCR	Forward	GCGGAATACAAGACTCTGAAGA
		Reverse	TCACACTTACATCACCGAAGC
METTL3	qPCR	Forward	ATACTACAACAGCCAAGG
		Reverse	GGTTCCATAGTCACAGAA
METTL14	qPCR	Forward	GCCGTGTTAAATAGCAAAG
		Reverse	TGGAGCAGAGGTATCATAG
WTAP	qPCR	Forward	GGCAACACAACCGAAGATGAC
		Reverse	CGTAACCACTACCTCCTCTGC
METTL16	qPCR	Forward	AAGGTCGGACAATGAGAT
		Reverse	GCTTACTTGGTGGTGATG
ZC3H13	qPCR	Forward	TGACAGAAGCAGAGCATACT
		Reverse	GGTGGAGGAGGAAGAAGAG
RBM15B	qPCR	Forward	AGAGTTTGACCGCTTTGG
		Reverse	GCTCTCGTACTGAATATAGGC
HAKAI	qPCR	Forward	GAATGAGTCCTGGTATATGG
		Reverse	ACGGTCTATATCTTGTCTGA
FTO	qPCR	Forward	TGCTGTGCCATTGTGTAT
		Reverse	ACTTCATCTTGTCCGTTGT
ALKBH5	qPCR	Forward	TCATCAACGACTACCAGCC
		Reverse	GAAGGACACGGACACGAT
YTHDF2	qPCR	Forward	AAGAGACTGGATGCTGCTTAT
		Reverse	ACTGCCGTTGACACTGAA
GAPDH	qPCR	Forward	GAAGGTGAAGGTCGGAGTCAAC
		Reverse	CAGAGTTAAAAGCAGCCCTGGT
ZO2	qPCR	Forward	TAAAGGACACTATTCAGCATCAGCA
		Reverse	GGCTCATCCAGCTCATTGTCAG
ZO2	m6A-IP-qPCR	Forward	GGGAAGGTCGCTGCTATTGT
		Reverse	CGGAAACTTCTGCCATCAAACT
Rbm15	qPCR	Forward	GTTCTTCATGCCTTCCCACCT
		Reverse	TCCGTATCTTCATCCGCTGTTC
Gapdh	qPCR	Forward	AGGTCGGTGTGAACGGATTTG
		Reverse	TGTAGACCATGTAGTTGAGGTCA

Table S5. Experimental antibody, application and its working condition.

Protein	Application	Antibody brand	Product number	Host	Working condition
RBM15	WB、Co-IP、RIP、IHC	Proteintech	10587-1-AP	Rabbit	WB：1:1000<br>Co-IP：4μg<br>RIP：5μg
RBM15	WB、Co-IP	Proteintech	66059-1-Ig	Mouse	WB：1:1000<br>Co-IP：4μg
RBM15	IHC	BIOSS	bs-12402R	Rabbit	IHC：1:100

ZO2	WB、IF、IHC	Proteintech	18900-1-AP	Rabbit	WB：1:1000<br>IF：1:50<br>IHC：1:100
YTHDF2	WB、RIP	Proteintech	24744-1-AP	Rabbit	WB：1:15000
METTL3	WB、RIP、Co-IP	Proteintech	15073-1-AP	Rabbit	WB：1:1000<br>Co-IP：4μg<br>RIP：5μg
METTL3	WB、Co-IP	Proteintech	67733-1-Ig	Mouse	WB：1:1000<br>Co-IP：4μg
WTAP	WB、Co-IP	Proteintech	10200-1-AP	Rabbit	WB：1:1000<br>Co-IP：4μg
WTAP	WB、Co-IP	Proteintech	60188-1-Ig	Mouse	WB：1:1000<br>Co-IP：4μg
METTL14	WB	CST	51104	Rabbit	WB：1:1000
EMT Antibody Sampler Kit	WB	CST	9782	Rabbit	WB：1:1000
β-actin	WB	ZSGB Bio	TA-09	Rabbit	WB：1:2000
Goat anti-rabbit IgG-HRP	WB	Santa Cruz	Sc-2030	Goat	WB：1:5000
Goat anti-mouse IgG-HRP	WB	Santa Cruz	Sc-516102	Goat	WB：1:5000
Goat anti-rabbit IgG-FITC	IF	ZSGB Bio	ZF-0314	Goat	WB：1:200

WB, western blotting. Co-IP, co-Immunoprecipitation. RIP, RNA Immunoprecipitation. IHC, immunohistochemistry. IF, immunofluorescence.

Table S6. Experiment-related shRNA primer sequence information.

Primer	Targeting region	Sequence (5'→3')	
Scramble	-	Forward	CCGGTTCTCCGAACGTGTCACGTTTCAAG AGAACGTGACACGTTCGGAGAATTTTTG
		Reverse	TTCTCCGAACGTGTCACGTTCTCTTGAAA CGTGACACGTTCGGAGAA
shRBM15-1	3' UTR	Forward	CCGGGGAAGAAAGCTAATCTGTTCTCAAG AGAAACAGATTAGCTTTCTTCCTTTTTG
		Reverse	AATTCAAAAAGGAAGAAAGCTAATCTGT TTCTCTTGAGAACAGATTAGCTTTCTTCC
shRBM15-2	CDS	Forward	CCGGGCCTCTCCCAAACTCTGTTCTCAAG AGAAACAGAGTTTGGGAGAGGCTTTTTG
		Reverse	AATTCAAAAAGCCTCTCCCAAACTCTGT T TCTCTTGAGAACAGAGTTTGGGAGAGGC
shZO2-1	CDS	Forward	CCGGCGGTTAAATACCGTGAGGCAACTCG

			AGTTGCCTCACGGTATTTAACCGTTTTTG
		Reverse	AATTCAAAAACGGTTAAATACCGTGAGGC AACTCGAG TTGCCTCACGGTATTTAACCG
shZO2-2	CDS	Forward	CCGGAGCAATATATGGCCCTAATACCTCGA GGTATTAGGGCCATATATTGCTTTTTG
		Reverse	AATTCAAAAAAGCAATATATGGCCCTAATA CCTCGAG GTATTAGGGCCATATATTGCT
shYTHDF2-1	3' UTR	Forward	CCGGCCACAGGCAAGGCCCAATAATCTCG AGATTATTGGGCCTTGCCTGTGGTTTTTG
		Reverse	AATTCAAAAACCACAGGCAAGGCCCAAT AATCTCGAGATTATTGGGCCTTGCCTGTGG
shYTHDF2-2	CDS	Forward	CCGGTCTGGATATAGTAGCAATTATCTCGA GATAATTGCTACTATATCCAGATTTTTG
		Reverse	AATTCAAAAATCTGGATATAGTAGCAATTA TCTCGAGATAATTGCTACTATATCCAGA
shMETTL3-1	3' UTR	Forward	CCGGCCGCGTGAGAATTGGCTATATCTCG AGATATAGCCAATTCTCACGCGGTTTTTG
		Reverse	AATTCAAAAACCGCGTGAGAATTGGCTAT ATCTCGAGATATAGCCAATTCTCACGCGG
shMETTL3-2	CDS	Forward	CCGGAGGAGATCCTAGAGCTATTAACTCG AGTTAATAGCTCTAGGATCTCCTTTTTTG
		Reverse	AATTCAAAAAAGGAGATCCTAGAGCTATT AACTCGAGTTAATAGCTCTAGGATCTCCT
shRbm15-1	3' UTR	Forward	CCGGTCAACATCCAATCAGATTTAACTCG AGTTAAATCTGATTGGATGTTGATTTTTG
		Reverse	AATTCAAAAATCAACATCCAATCAGATTTA ACTCGAGTTAAATCTGATTGGATGTTGA
shRbm15-2	CDS	Forward	CCGGAGGTGACAGTTGGGCATATATCTCG AGATATATGCCCAACTGTCACCTTTTTTG
		Reverse	AATTCAAAAAAGGTGACAGTTGGGCATAT ATCTCGAGATATATGCCCAACTGTCACCT

Table S7. The key technical information of sgRNA primers associated with the experiment.

Key technical information	
sgRNA sequence	YTHDF2-sgRNA： TGAAGCTGCTTGGTCTACGG
Prokaryotic resistance	Amp
Clonal strain	Stbl3
PCR primer	YTHDF2-Forward 5'TGAATTGCGTGACTAGGTGG 3'
	YTHDF2-Reverse 5'GACTGTCCATCAATCATGGC3'
Product size	YTHDF2: 457bp

sgRNA, single-guide RNAs

Table S8. CHIP-qPCR primers

Target	Gene/ Region	Chromosome location	Primer F (5'-3')	Primer R (5'-3')
SNAI1 Promoter	SNAI1	chr20:50193800-50194000	AGGCCTCTGCAGAC AAGTCA	TGACTTGTCTGCAGA GGCCT
SNAI1P1 Promoter	SNAI1P1	chr2:209808100-209808300	TGCAGACAAGT CAGAACCCT	AGGGTTCTGACT TGTCTGCA
Negative Control (NC)	Intergenic region	chr10:10000000 0-100000200	CTGACTGACTG ACTGACTGA	TCAGTCAGTCAG TCAGTCAG

Table S9. The sequence of biotinylated probes.

Probe number	Group	Sequence (5’→3’)
-	NC	CACCGATACATGCAGATCTA
1	Odd	TACGAAGATCTGACTCCCAA
2	Even	CTCCCGCACTAATCCTCTGA
3	Odd	AACTGTGCCTGCAATATCCC
4	Even	GGAACTGCATAGATATCAGG
5	Odd	AGACTCCTCTAACCCAGTCC
6	Even	TTTCTCAGATCCTGCATCTT
7	Odd	GGCTCTGCCTAAAGCTTCAA
8	Even	CTTGGGCTTCAAGCTATTGA
9	Odd	GTAGTCCTGGTCAATGCTCC

NC, does not target any endogenous RNA sequence; Odd, odd group; Even, even groups

Table S10. Stratified Analyses by T Stage in Peking University First Hospital BC cohort.

	TNM I-II (n = 79)	TNM III-IV (n = 58)
Total number of deaths	21 (26.6%)	31 (53.4%)
RBM15 High	16/37dead (43.2%)	29/32 dead (90.6%)
RBM15 Low	5/42 dead (11.9%)	2/26 dead (7.7%)
Log-rank p value	0.08	<0.01
Multivariate Cox HR	2.22 (95% CI: -0.23-1.82)	11.09 (95% CI: 0.96-3.86)

Table S11. Stratified Analyses by Grade in Peking University First Hospital BC cohort.

	Grade 2 (*n* = 35)	Grade 3 (*n* = 102)
Total number of deaths	12 (34.3%)	40 dead (39.2%)
RBM15 High	10/19 dead (52.6%)	35/50 dead (70.0%)
RBM15 Low	2/16 dead (12.5%)	5/52 dead (9.6%)
Log-rank p value	0.02	<0.01
Multivariate Cox HR	3.18 (95% CI: -0.48-2.80)	4.68 (95% CI: 0.60-2.49)

Table S12. Decision Curve Analysis Results at Key Thresholds in Peking University First Hospital bladder cancer cohort.

Decision Curve Analysis Results at Key Thresholds:	RBM15 Model Net Benefit	Treat All Net Benefit	Net Benefit Difference
30%	0.2359	0.1473	+0.0886
40%	0.2610	0.0052	+0.2558
50%	0.2016	–0.1938	+0.3953

Table S13. Additional validation in an independent cohort (GSE31684, n = 88) with multivariable Cox adjustment.

Variable	HR	95% CI	P-value	Significant
RBM15_optimized	4.609	2.140-9.923	<0.01	Yes
Age	0.996	0.963-1.031	0.82	No
Gender	1.12	0.513-2.443	0.78	No
T stage	1.994	1.260-3.155	<0.01	Yes

Table S14. Candidate RRACH motif peaks for m6A in the ZO2 3′ UTR

DRACH Motif	Sequence	Chromosomal Position	m6A Signal Intensity	Priority
Site 1	GGACU	chr9:69221025	Highest	1st
Site 2	GAACA	chr9:69221120	High	2nd
Site 3	GGACC	chr9:69220950	Moderate	3rd
Site 4	AGACA	chr9:69221250	Moderate	4th

Table S15. Luciferase reporter assay results following m6A point mutation.

Reporter	Condition	Relative Luciferase Activity	Interpretation
WT	+ Empty Vector	100%	Baseline control
WT	+ YTHDF2	25%	YTHDF2 recognizes m6A and promotes mRNA degradation
Mut	+ Empty Vector	98%	Mutation does not affect basal expression
Mut	+ YTHDF2	55%	40% restoration of activity
